# Advances in Optical Nanoprobes for Bacterial Infection Diagnosis

**DOI:** 10.34133/research.1466

**Published:** 2026-10-29

**Authors:** Minzhen Wen, Yejiao Shi, Xinning Wang, Hao Li, Caiping Yin, Mingyi Sun, Tong Xu, Houbing Zhang, Yan Bao, James P. Basilion, Xi Liu, Menghua Xiong, Dong Luo

**Affiliations:** ^1^ School of Biomedical Sciences and Engineering, South China University of Technology, Guangzhou International Campus, Guangzhou, Guangdong, China.; ^2^ National Engineering Research Centre for Tissue Restoration and Reconstruction, South China University of Technology, Guangzhou, Guangdong, China.; ^3^ Institute of Translational Medicine, Shanghai University, Shanghai, China.; ^4^ Department of Biomedical Engineering and Department of Radiology, Case Western Reserve University, Cleveland, OH, USA.; ^5^ Guangdong Province Key Laboratory of Biomedical Engineering, South China University of Technology, Guangzhou, Guangdong, China.; ^6^ Guangdong Provincial Key Laboratory of Malignant Tumor Epigenetics and Gene Regulation, Guangdong-Hong Kong Joint Laboratory for RNA Medicine, Medical Research Center, Sun Yat-Sen Memorial Hospital, Sun Yat-Sen University, Guangzhou, Guangdong, China.; ^7^ Medical Innovation Research Department, PLA General Hospital and PLA Medical College, Beijing, China.; ^8^ Key Laboratory of Biomedical Materials of the Ministry of Education, South China University of Technology, Guangzhou, Guangdong, China.

## Abstract

Bacterial infections represent a major threat to global public health, and the rising prevalence of antibiotic resistance underscores the urgent need for early and accurate diagnosis. Optical imaging technologies, characterized by high sensitivity, spatiotemporal resolution, real-time monitoring capability, and noninvasiveness, have emerged as promising complementary and alternative approaches for bacterial infection diagnosis. This review comprehensively summarizes recent advances in optical nanoprobes for diagnosing bacterial infections. We first outline the structural and metabolic distinctions between bacteria and mammalian cells, along with the dynamic features of the infection microenvironment, which collectively provide a biological foundation for selective targeting and infection-specific imaging. We then detail the working principles, design strategies, and characteristics of various optical nanoprobes, including fluorescence, chemiluminescence, bioluminescence, photoacoustic, and Raman imaging probes, highlighting their applications in bacterial detection. Finally, we focus on in vitro diagnostic scenarios, categorizing and evaluating the performance, advantages, and challenges of optical nanoprobe-based technologies across clinical specimens such as blood, urine, and sputum. By offering a systematic perspective, this review aims to provide actionable insights for clinical translation and to promote the development of next-generation rapid diagnostic platforms for bacterial infections.

## Introduction

Bacterial infection is one of the major threats to global public health [1–3]. As common pathogens, bacteria can invade the human body via various routes, causing diseases such as pneumonia, osteomyelitis, wound infections, and sepsis [4,5]. Improper treatment can also lead to multiple complications and even threaten life. Although humans have been able to effectively control bacterial infections since the advent of penicillin in 1929 [6], the diagnosis and treatment of bacterial infections have become increasingly difficult due to the continuous increase in antibiotic resistance [7–9]. According to a report by the World Health Organization, about 1.27 million people died from bacterial infections worldwide in 2019, accounting for 13.6% of all global deaths [10]. Meanwhile, *The Lancet* predicts that by 2050, 10 million people will die each year from antibiotic-resistant diseases globally, far exceeding the number of deaths caused by cancer [11].

One of the current difficulties hindering the effective treatment of bacterial infectious diseases is the lack of precise diagnostic techniques in the early stage of bacterial infection [12–14]. Early diagnosis can reduce the risk of systemic infection, avoid life-threatening complications such as sepsis, and provide important clinical guidance to help doctors formulate more accurate and effective treatment plans. Molecular imaging is an emerging interdisciplinary technology that integrates medical imaging with molecular biology, materials science, and bioengineering. It enables complex biological processes, such as gene expression and intracellular signaling, to be visualized as intuitive images, providing spatial, qualitative, and quantitative information on disease initiation and progression at both anatomical and molecular levels [15,16]. By linking molecular events to anatomical structures, molecular imaging offers a precise foundation for disease staging and facilitates early and accurate diagnosis [17,18]. The development of in vivo and in vitro bacterial imaging probes is an important prerequisite for the early detection of bacterial infections and is also one of the important research directions in the current field of molecular imaging.

The current clinical gold standard for infection diagnosis is microbial culture [19–21]. However, this method suffers from a long detection cycle, limited sensitivity, and high operator expertise requirements. Meanwhile, slow-growing obligate aerobic bacteria such as *Mycobacterium tuberculosis* require prolonged culture in vitro, which may cause diagnostic delays [22]. In addition, clinical samples (e.g., blood, urine, feces, or cerebrospinal fluid) can be analyzed using molecular techniques such as immunoassays and polymerase chain reaction (PCR). Although these methods have high sensitivity, they are costly, prone to contamination, and unable to distinguish live and dead bacteria and usually require a long pre-culture period [23]. For some deep tissue bacterial infections, invasive biopsy is usually required. However, biopsy has certain risks and may lead to secondary infection. In clinical practice, some imaging examination methods are also often used, such as computed tomography (CT) and magnetic resonance imaging (MRI). They have advantages in locating the infection site, but they lack specificity and are difficult to distinguish between aseptic inflammation and bacterial infection [24], and cannot determine the bacterial load and species.

In contrast, optical imaging technology is widely regarded as a promising complementary and alternative approach, characterized by its high sensitivity, high spatiotemporal resolution, real-time dynamic monitoring capability, and noninvasive or minimally invasive nature [25–27]. The core principle relies on the interaction between photons and biological tissues or specific targets, enabling the visualization of spatial distribution and metabolic activities of bacteria by detecting emitted, scattered, or absorbed optical signals [28,29]. As key components of this technology, optical nanoprobes are molecular or nanoscale materials capable of interacting with specific bacterial related biomarkers, such as enzymes, bacterial surface structures, or metabolites, through specific recognition mechanisms (e.g., enzyme–substrate reactions, antigen–antibody binding, and aptamer–target interactions) [30]. These probes convert such specific interaction into detectable optical signals, such as fluorescence [31], chemiluminescence [32], or surface-enhanced Raman scattering (SERS) [33]. By transforming biological information into high signal-to-noise optical readouts, they allow for specific and highly sensitive bacterial detection and imaging, even within complex biological matrices [34]. Consequently, optical imaging probes have increasingly become a research focus, particularly in the rapid detection of clinical samples such as blood or urine, demonstrating considerable potential for point-of-care diagnostic applications (Fig. 1).

**Fig. 1.**
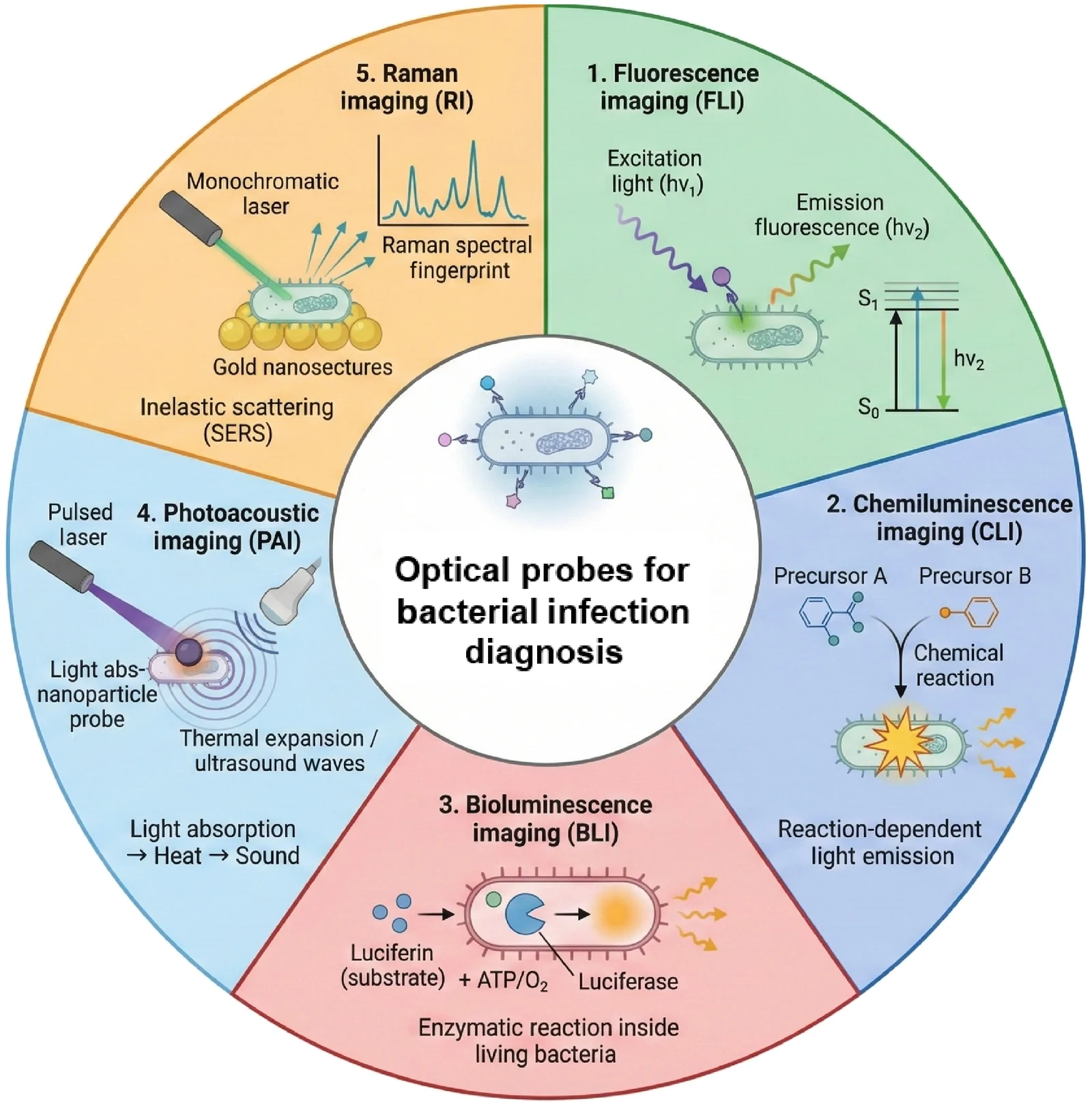
Overview of imaging strategies for optical nanoprobes in bacterial infection diagnosis.

This review aims to summarize the latest research advances in optical nanoprobes for the diagnosis of bacterial infections in recent years. We first summarize the structural and metabolic characteristics that distinguish bacteria from mammalian cells, together with the unique features of the bacterial infection microenvironment. These distinctive properties form the biological basis for selective bacterial targeting and infection-specific imaging. Then, we provide a detailed introduction to the working principles, design strategies, and characteristics of various types of optical probes for bacterial detection, including fluorescence probes, chemiluminescence probes, bioluminescence probes, Raman imaging probes, and photoacoustic imaging (PAI) probes. Subsequently, we focus on in vitro diagnostic applications, categorizing and evaluating the current research status, advantages, and challenges of optical probe-based diagnostic technologies across different sample matrices, such as blood, urine, sputum, and other common clinical specimens. Ultimately, by offering a comprehensive perspective in this field, this review seeks to provide actionable insights for clinical applications and promote the development of next-generation rapid diagnostic technologies for bacterial infections.

## Bacteria and Infection Microenvironment

Bacterial infection is a dynamic and evolving interaction between pathogen and host, strongly shaped by the intrinsic properties of the microorganism and the local infection microenvironment. Microbial factors such as surface architecture, metabolic activity, virulence determinants, and adaptive stress responses govern adhesion, invasion, immune evasion, and colonization [35]. These microbial activities rapidly remodel the surrounding microenvironment, altering nutrient availability, pH, oxygen tension, and host signaling pathways [36]. In parallel, the host mounts coordinated innate and adaptive immune responses, including physical and chemical barriers, cellular defenses, inflammatory cascades, and tissue remodeling, which further modify local conditions [37]. The continuous feedback between microbial traits and microenvironmental cues drives pathogen adaptation and persistence while influencing immune effectiveness. Together, these tightly regulated and reciprocal processes define the biological landscape at the site of infection, ultimately determining disease progression, clinical outcomes, and the success of therapeutic interventions.

The structural specificity of the pathogen forms the basis of its pathogenicity [38]. Gram-positive organisms, such as *Staphylococcus aureus*, are characterized by a thick (20 to 80 nm), multilayered peptidoglycan matrix interlaced with anionic polymers, including lipoteichoic acid (LTA) and wall teichoic acid (WTA) [39]. These cell wall components impart a strongly negative surface charge and play essential roles in maintaining ionic homeostasis, mediating host adhesion, and modulating host–pathogen interactions [40,41]. Gram-negative bacteria, such as *Escherichia coli*, have a thin (2 to 7 nm) peptidoglycan layer enclosed by an asymmetric outer membrane [42]. Its outer leaflet is rich in lipopolysaccharide (LPS), whose variable O-antigen defines serotypes, while the inner leaflet contains phospholipids [43]. The lipid A component of LPS is a potent endotoxin responsible for triggering severe systemic inflammatory responses, including sepsis [44].

Beyond the cell wall, many pathogens produce an outer capsule or slime layer, such as *Streptococcus pneumoniae*’s hyaluronic acid capsule or *Pseudomonas aeruginosa*’s alginate, which can reach hundreds of nanometers in thickness and protects against phagocytosis and complement [45]. Surface appendages like flagella (flagellin) and pili (e.g., type I pili) support motility and adhesion while serving as pathogen-associated molecular patterns (PAMPs) recognized by host immune receptors [46]. Beyond the cell walls and capsules, the fundamental phospholipid composition of bacterial cytoplasmic membranes exhibits profound differences from that of mammalian host cells, representing another layer of structural specificity. Bacterial membranes are predominantly composed of phospholipids such as phosphatidylglycerol (PG), phosphatidylethanolamine (PE), and cardiolipin (CL), with their head groups often carrying a net negative charge [47,48]. In contrast, mammalian cell membranes are rich in zwitterionic phospholipids like phosphatidylcholine (PC) and sphingomyelin, which maintain overall charge neutrality under physiological conditions [49]. The prevalent negative charge on bacterial surfaces, arising from both anionic membrane lipids and cell wall polymers like teichoic acids and LPS, contrasts sharply with the more neutral or selectively charged patches on mammalian cell membranes [50]. This fundamental biochemical distinction provides a potential basis for differential electrostatic interactions, which can be exploited for selective targeting [51].

The sustained growth, replication, and structural integrity of bacteria depend on a highly conserved metabolic network that is fundamentally distinct from that of mammalian host cells. Central to this distinction is the biosynthesis of peptidoglycan, a process absent in eukaryotes. Peptidoglycan assembly involves precise cross-linking steps catalyzed by penicillin-binding proteins (PBPs), using d-alanine residues carried by the precursor UDP-MurNAc-pentapeptide [52]. In contrast, mammalian cells lack both peptidoglycan and the enzymes required for its synthesis, rendering these pathways uniquely bacterial. Similarly, the production of surface polysaccharides, including capsules and O-antigens, follows specialized bacterial routes such as the Wzx/Wzy-dependent assembly pathway [53]. Importantly, many of these bacterial metabolic processes display relative substrate flexibility, permitting the incorporation of structurally analogous exogenous molecules, such as non-natural d-amino acid analogs or modified monosaccharides, into newly synthesized cell walls or extracellular polymers [54]. This metabolic uniqueness highlights key bacterial–host differences and enables targeted imaging, selective antimicrobials, and precise probing of infection sites.

Beyond static structures and core metabolism, bacterial collective behaviors further increase the complexity of the infectious milieu. Many pathogenic bacteria rely on quorum sensing (QS), a cell–cell communication system mediated by secreted autoinducer molecules (e.g., acyl-homoserine lactones in Gram-negatives and autoinducing peptides in Gram-positives), to coordinate population-wide gene expression [55]. This communication mechanism acts as a central switch for biofilm formation. Biofilms are highly organized microbial communities encased in a self-produced extracellular polymeric matrix [56]. This matrix, primarily composed of exopolysaccharides [e.g., polysaccharide intercellular adhesin (PIA) in *S. aureus* and alginate in *P. aeruginosa*], extracellular DNA, proteins (including amyloid fibers), and lipids, creates a diffusion-limited barrier that protects resident bacteria [57]. This spatial architecture gives rise to pronounced chemical gradients within the biofilm. Regions at the biofilm periphery are typically well oxygenated and metabolically active, whereas limited diffusion toward the interior results in hypoxic or fully anaerobic conditions in the core. Concurrent accumulation of metabolic byproducts further acidifies the interior, with local pH values dropping to ~5.5 or lower [58]. Beyond structural components, bacteria in biofilms and invasive infections secrete a diverse array of exoenzymes that actively degrade host tissues. These include hyaluronidases that break down hyaluronic acid, proteases (such as *S. aureus* V8 protease and *P. aeruginosa* elastase) that cleave host structural and immune proteins, and lipases that disrupt host cell membranes [59].

Concurrently, the host immune response engages dynamically with the pathogen to generate a highly heterogeneous infection microenvironment [60]. Following early immune activation, large numbers of neutrophils and macrophages are recruited to the site of infection [61]. Macrophages further differentiate into pro-inflammatory (M1) or anti-inflammatory (M2) phenotypes, thereby shaping the local inflammatory balance [62]. Intense cellular respiration, oxidative burst activity, and frequent microcirculatory dysfunction collectively drive progressive hypoxia and pronounced extracellular acidification at the infection site, with pH values typically ranging from ~5.5 to 6.5 due to the accumulation of lactic acid and fatty acids [63,64]. In parallel, inflammatory mediators such as histamine, bradykinin, and vascular endothelial growth factor (VEGF) compromise tissue barrier integrity, leading to increased vascular permeability, edema, and extracellular matrix degradation mediated by up-regulated host proteases, including matrix metalloproteinases (MMPs) [65].

Importantly, these target classes convey different diagnostic information with respect to bacterial viability and burden. Metabolic-incorporation strategies generally depend on ongoing biosynthesis and therefore preferentially report viable or metabolically active bacteria; in contrast, probes recognizing relatively persistent bacterial surface structures or physicochemical features may detect both viable and nonviable cells, whereas enzyme-activated probes primarily report target-enzyme activity rather than viability per se, and probes responsive to pH, reactive oxygen species (ROS), hypoxia, or other microenvironmental cues reflect infection-associated signals rather than bacterial burden itself.

In summary, the distinctive structural features of bacteria, together with the dynamically evolving infection microenvironment across molecular, cellular, and tissue scales, clearly differentiate bacterial infections from normal physiological conditions and sterile inflammation. These pathogen-specific architectures, metabolic pathways, collective behaviors, and host–pathogen-driven microenvironmental cues form a multidimensional and highly specific set of targets. Collectively, they provide a robust biological foundation for the rational design of precise optical imaging probes capable of selectively detecting and delineating bacterial infections (Fig. 2).

**Fig. 2.**
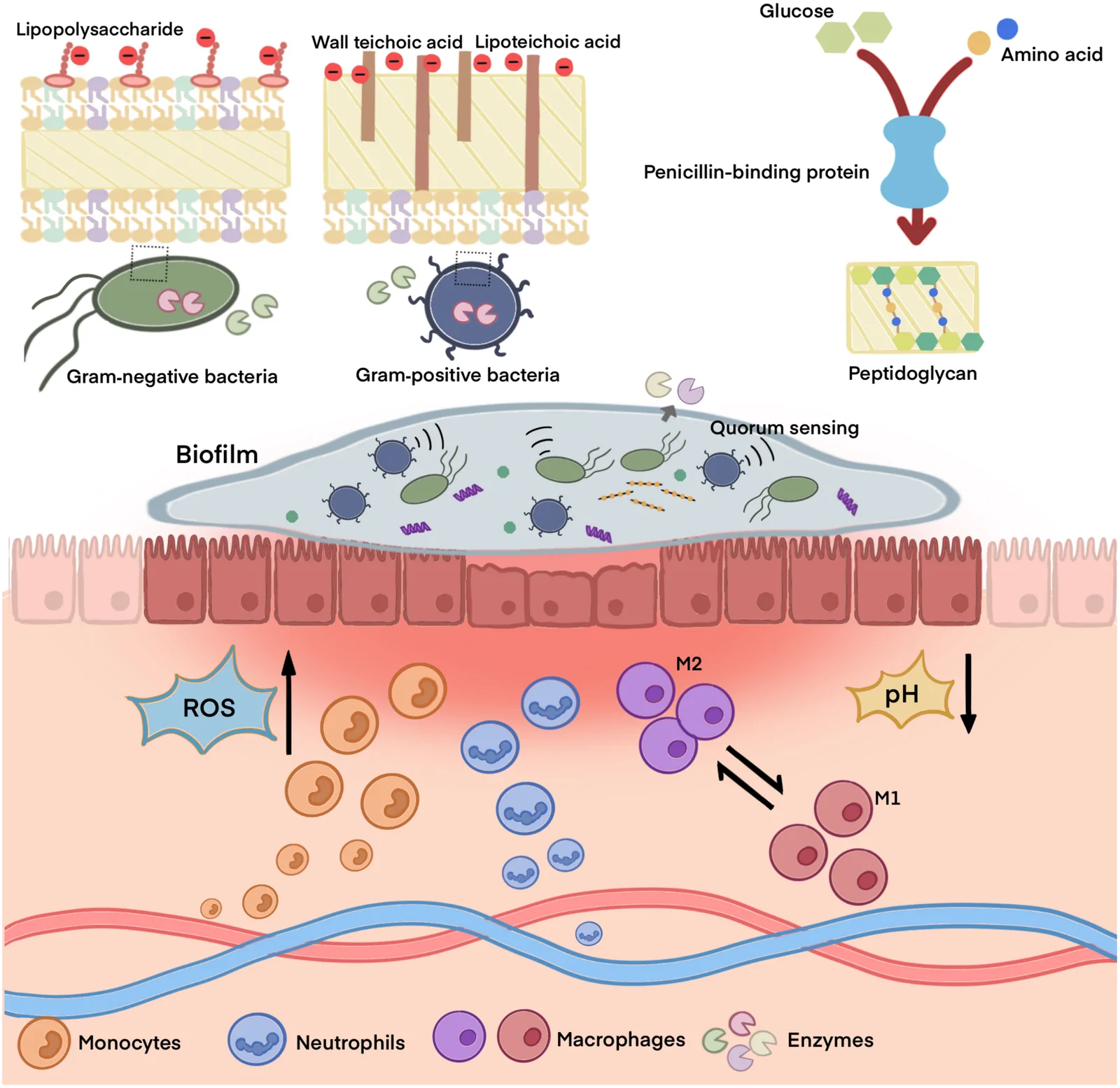
Schematic illustration of the unique structural and metabolic features of bacteria and the infection microenvironment, which provide a biological foundation for selective targeting and infection-specific imaging.

## Optical Nanoprobes for Bacterial Infection Diagnosis

### Fluorescence imaging

Fluorescence imaging of bacterial infection is a noninvasive optical technique that visualizes bacteria in vivo by detecting light emitted from fluorescent probes [66]. These probes may be genetically encoded (e.g., bacteria engineered to express fluorescent proteins) or externally administered dyes, nanoparticles, or targeted molecules that selectively bind to bacterial components such as cell-wall structures, metabolic enzymes, or virulence factors. When illuminated with excitation light, the probes emit fluorescence signals that can be captured with high sensitivity using specialized imaging systems [67]. According to the types of fluorescent materials used, probes for detecting bacterial infections can be broadly categorized into organic fluorescent probes and inorganic nanoprobes. Organic fluorescent probes offer precise control over their physicochemical properties because their molecular structures can be easily modified to adjust parameters such as photostability, emission wavelength, hydrophobicity, and targeting specificity. These probes can be engineered to respond to bacterial metabolites, enzymes, or microenvironmental changes, enabling highly sensitive and selective detection [68]. In contrast, inorganic nanoprobes such as up-converting nanoparticles, quantum dots (QDs), carbon dots (CDs), and metal nanoclusters (MNCs) provide superior brightness, high photostability, and tunable optical properties, making them suitable for deep-tissue imaging and long-term tracking [69]. Together, these classes of fluorescent materials provide versatile platforms for accurate, real-time visualization of bacterial infections.

#### Organic fluorescent nanoprobes

Organic small-molecule fluorescent dyes are widely recognized as powerful tools for bacterial detection and imaging due to their high sensitivity, good biocompatibility, and ease of structural modification. These features allow the creation of probes with tailored optical properties and targeting capabilities, enabling rapid and accurate visualization of bacterial infections [70]. Commonly used small-molecule fluorescent dyes, including fluorescein [71], coumarin [72], rhodamine [73], boron–dipyrromethene (BODIPY/fluoroborane) [74], phycocyanin [75], eosin [76], and TCF (2-dicyanomethylene-3-cyano-4-methyl-4*H*-chromene) derivatives [77], offer diverse excitation/emission profiles, tunable photophysical behavior, and robust chemical stability, making them suitable platforms for constructing bacteria-responsive fluorescent probes [78]. Researchers have developed a wide range of fluorescent probes that respond to diverse targets associated with bacterial infections, such as pH changes, bacterial enzymes, surface receptors, and ROS [79]. Such stimulus-responsive designs allow for enhanced specificity, real-time monitoring, and improved accuracy in visualizing bacterial infections [40].

##### Infection diagnosis based on bacterial membrane structure

Unlike mammalian cells, bacteria possess a highly organized and chemically distinctive wall composed of conserved biomacromolecules such as peptidoglycan, teichoic acids (Gram-positive), and LPS (Gram-negative) [80]. These components provide structural integrity and present bacteria-exclusive molecular motifs that fluorescent probes can selectively target with minimal interference from host tissues [81]. Based on this, Kwon et al. [82] developed a universal fluorescent probe, BacGO, based on a boronic acid recognition motif that selectively binds sugar components in the peptidoglycan-rich cell walls of Gram-positive bacteria. In a corneal infection model, BacGO enabled clear imaging of *S. aureus* infections while showing minimal fluorescence in *P. aeruginosa*-infected tissues. Similarly, Zhao et al. [83] reported a nanoprobe (HF-D-Ala NPs) that was formed by self-assembly of amphiphilic fluorescent d-alanine derivatives. In metabolically active bacteria, d-alanine incorporation induces nanoparticle disassembly and cell-wall integration of HF-D-Ala, triggering a fluorescence “turn-on” signal and enabling highly specific bacterial detection and imaging (Fig. 3A and B).

**Fig. 3.**
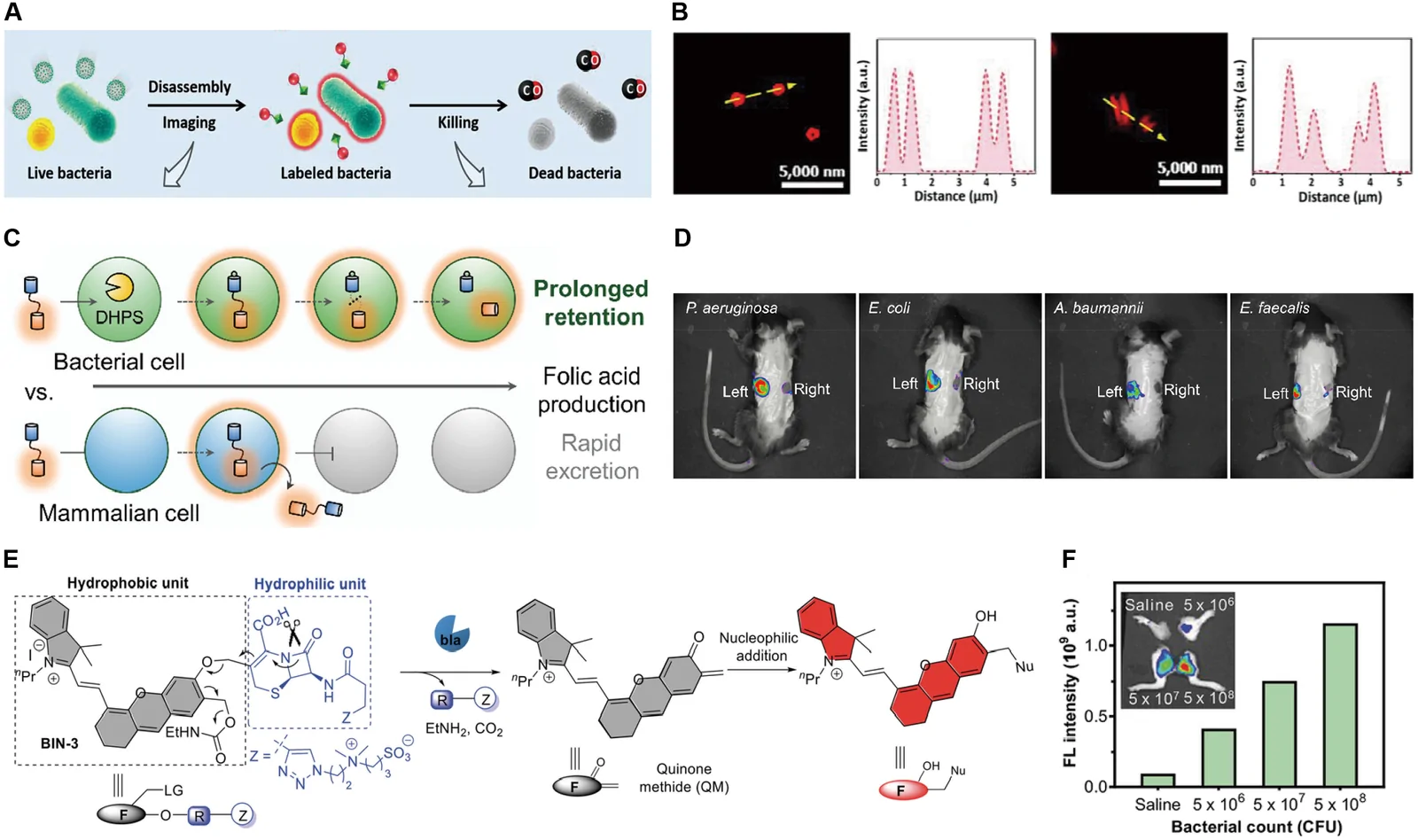
Design strategies of organic fluorescent probes for imaging bacteria and infection. (A) Labeling bacterial wall with HF-D-Ala NPs based on amphiphilic fluorescent d-alanine and subsequent CO-mediated killing under visible light [83]. Copyright 2022, Wiley. (B) Representative fluorescence images (scale bar, 5 μm) and corresponding intensity analysis of *S. aureus* and *E. coli* labeled with HF-D-Ala NPs*.* Reproduced with permission [83]. Copyright 2022, Wiley. (C) Schematic illustration of bacteria-specific imaging via DHPS-dependent metabolic retention: PABA-DCM is internalized and retained in DHPS-high bacteria, while rapidly cleared from mammalian cells lacking DHPS [92]. (D) In vivo fluorescence imaging of implant-related wound infection models treated with PABA-DCM@GO. Left wound: viable bacteria; right wound: heat-killed bacteria [92]. Copyright 2022, American Chemical Society. (E) Resistant bacteria imaging based on β-lactamase-expressing pathogens using BIN-3 probe, which undergoes a hydrophilic-to-hydrophobic conversion and binds to nucleophilic residues within bacterial cells [98]. (F) In vivo fluorescence imaging of a mouse 5 h post-administration of BIN-3, with quantified signal intensities of thigh regions shown in the adjacent bar graph [98]. Copyright 2025, Wiley.

For detecting intracellular bacteria or imaging within complex infection microenvironments, more precise structural-targeting designs are required. One promising approach involves using bacteriophages, which naturally recognize bacterial cell walls, to achieve selective labeling of intracellular pathogens. For instance, Zhang et al. [84] developed a multifunctional nanoconjugate (MS2-DNA-AIEgen) based on an AIEgen–bacteriophage–DNA hybrid system for the specific recognition, imaging, and eradication of intracellular bacterial infections. Its strategy integrates the intrinsic targeting specificity of the bacteriophage (MS2) toward its host bacteria, such as *E. coli* ATCC15597 and its drug-resistant strain. Upon cellular internalization, the probe enables precision localization of intracellular bacteria under phage guidance, while the aggregation-induced emission photosensitizer (AIEgen) accumulates on the bacterial surface and emits strong near-infrared (NIR) fluorescence for imaging. The system achieves a high imaging performance, exhibiting near-perfect labeling efficiency, excellent specificity against nonhost bacteria, robust photostability, and fluorescence saturation within approximately 40 min of incubation. Simultaneously, the process incorporates a concurrent photodynamic bactericidal capability, realizing an integrated theranostic platform that combines targeted recognition, fluorescence imaging, and synergistic eradication of intracellular bacteria [84].

Beyond imaging-based bacterial recognition, nanomaterial-enabled strategies have also been developed to actively interfere with bacterial pathogenesis. For instance, Gao et al. [85] reported a nano-enabled quenching strategy that catalytically disrupts bacterial cell–cell communications, effectively preventing biofilm formation by intercepting QS signaling. This approach highlights the potential of nanomaterials not only as imaging probes but also as therapeutic agents that target bacterial social behaviors. Beyond disrupting bacterial communication, direct targeting of bacterial membrane phospholipids offers another therapeutic route [86].

##### Infection diagnosis based on bacterial membrane charge

Bacterial membranes possess an inherently negative surface charge arising from the abundance of anionic polysaccharides and phospholipids [87]. To take advantage of this feature, Koirala et al. [88] developed a NIR fluorescent probe, BacVue, for imaging intracellular uropathogenic *E. coli* (UPEC) in urinary tract infections (UTIs). The probe combines a membrane-permeant zinc(II)-dipicolylamine derivative for bacterial targeting with a charge-neutral cyanine fluorophore. In a mouse UTI model, intravesical administration of BacVue enabled specific and persistent visualization of infected bladders, exhibiting 2.4-fold higher ex vivo fluorescence intensity compared to uninfected controls (*P* < 0.0001). Additionally, Long et al. [89] reported an imidazolium-derived pyrene compound (probe 1) that self-assembles into nanoparticles in aqueous solution, leading to fluorescence quenching via aggregation-caused quenching (ACQ). Upon interaction with negatively charged bacterial surfaces, the synergistic effect of electrostatic attraction and hydrophobic forces triggers disassembly of the probe, restoring pyrene fluorescence and resulting in a turn-on response. Simultaneously, the probe exhibits coexisting pyrene excimer and monomer emission on the bacterial surface, providing a ratiometric fluorescent signal (*I*_482_/*I*_375_) that enhances detection reliability and reduces interference. This probe not only enables discrimination between Gram-negative and Gram-positive bacteria but also allows construction of a 2-dimensional recognition map based on changes in fluorescence intensity and emission ratios, successfully distinguishing 6 different bacterial species.

##### Infection diagnosis based on bacterial metabolism

Bacteria possess unique biosynthetic processes that rely on specialized enzymes and metabolic pathways absent in mammalian cells, and fluorescent probes have been used to label metabolites involved in bacterial metabolism, such as sugars, amino acids, or nucleic acids [90]. As these labeled compounds are naturally incorporated into bacterial metabolic pathways, they can covalently or noncovalently bind to bacterial structures, enabling precise and efficient labeling [91]. PABA-DCM fluorescent probe developed by Zhong et al. [92] is a representative fluorescent probe for metabolic pathway targeting. This probe is based on para-aminobenzoic acid (PABA), a key precursor in the bacterial folic acid synthesis pathway, and is specifically recognized and metabolically incorporated by bacterial dihydropteridine synthase (DHPS). Experimental results demonstrate that PABA-DCM is taken up by both Gram-positive and Gram-negative bacteria and participates in their folic acid synthesis, whereas it is rapidly expelled by mammalian cells. Its ability to achieve specific fluorescence imaging of live bacterial infection has been successfully validated in a mouse wound infection model (Fig. 3C and D) [92].

##### Infection diagnosis based on infectious microenvironment

Infection sites are characterized by a distinctive pathological microenvironment, including acidic pH, elevated levels of bacteria-associated enzymes, and increased ROS [93]. By designing probes that respond to these infection-associated cues, researchers can achieve selective activation of fluorescence signals, minimizing background interference and enabling precise imaging of bacterial infections.

For instance, targeting strategies based on microenvironmental acidity have been developed to improve imaging specificity. Peng et al. [94] developed a pH-responsive targeted nanosystem, ZEI@GCS, based on persistent luminescence nanoparticles (ZGS) for high signal-to-noise ratio NIR imaging of bacterial infections. Targeting is achieved through glycol chitosan, which becomes positively charged in the slightly acidic infection microenvironment and electrostatically adheres to negatively charged bacterial surfaces [e.g., methicillin-resistant *S. aureus* (MRSA) and *E. coli*]. Following excitation with a 659-nm light-emitting diode (LED), persistent luminescence signals were collected at 700 nm, yielding a signal-to-noise ratio 18 times higher than that of indocyanine green (ICG). This system enables real-time monitoring of infection sites and assessment of therapeutic clearance.

Enzyme-responsive probes represent another major category. Nitroreductase (NTR), a member of the flavin enzyme family, is widely expressed in both bacterial and mammalian cells [95]. Its primary function is to catalyze the reduction of nitro compounds, converting nitro groups into nitrite or other nitrogen-containing products [95]. In bacteria and other microorganisms, NTR plays an important role in nitrogen metabolism and the nitrogen cycle [96]. Xiang et al. [97] developed an NTR-responsive copper-based nanoprobe, Cu_2_-Se@BSA@NTRP, for rapid in situ fluorescence imaging of bacterial infections. The probe releases the loaded NTRP molecules triggered by the weakly acidic microenvironment; once internalized into bacteria such as *E. coli* and *S. aureus*, NTRP is specifically reduced by the bacterially overexpressed endogenous NTR enzyme, activating a fluorescence “turn-on” response that enables specific recognition of viable bacteria. The system reaches peak fluorescence after 6 h of incubation with bacteria in vitro, while in vivo, topical application produces a strong signal in infected corneas within 30 min, offering an imaging window of 20 min to 2 h. This approach allows noninvasive, high-contrast fluorescence diagnosis and therapeutic monitoring of bacterial keratitis.

Chen et al. [98] developed a NIR fluorescent probe, BIN-3, which employs a dual strategy of hydrophilicity switching and self-immobilization to selectively target and image antibiotic-resistant bacterial infections expressing β-lactamase. Upon enzymatic hydrolysis by bacterial β-lactamase, BIN-3 undergoes a hydrophilic-to-hydrophobic conversion and generates a quinone methide intermediate that covalently binds to nucleophilic residues within bacterial cells, leading to localized fluorescence activation and substantial signal enrichment (>1,500-fold within bacteria). This design enables highly sensitive and specific detection of β-lactamase-expressing pathogens in vivo, with a demonstrated detection limit as low as 2 × 10^6^ colony-forming units (CFU) and a signal-to-background ratio (SBR) of up to 10 in murine models. The probe successfully visualizes infections in both subcutaneous abscess and myositis models, permitting real-time, noninvasive monitoring of bacterial burden (Fig. 3E and F) [98].

To further improve diagnosis accuracy, researchers have also developed a dual-targeting strategy [99]. Han et al. [100] developed a dual-channel fluorescent probe, Nap-CefTTPy, that leverages 2 distinct bacterial biomarkers for concurrent activation: NTR, which is highly expressed in both Gram-positive and Gram-negative bacteria, triggers green fluorescence through nitro-group reduction, while binding to PBPs on bacterial membranes restricts intramolecular motion and enhances red emission. This design enables crosstalk-free, dual-channel fluorescence imaging under single-wavelength excitation (488 nm). The probe was validated against a panel of clinically relevant bacterial strains, including Gram-positive *S. aureus* and *Enterococcus faecium*, as well as Gram-negative *Enterobacter cloacae*, *Klebsiella pneumoniae*, and *P. aeruginosa*. In a murine wound infection model with *S. aureus*, the probe allowed precise spatial mapping of infection sites through separate green (500 to 600 nm) and red (600 to 700 nm) emission channels.

#### Inorganic fluorescence nanoprobes

Inorganic fluorescence nanoprobes have advanced rapidly as robust platforms for imaging bacterial infections, owing to their exceptional photostability, tunable optical properties, and flexible surface chemistry [101]. Early QD-based probes provided high brightness but raised biocompatibility concerns, prompting the development of safer alternatives such as upconversion nanoparticles (UCNPs) [102], CDs, and MNCs [103]. Recent progress in surface engineering has enabled highly specific bacterial targeting through the incorporation of antibodies, peptides, antibiotics, bacteriophages, or metabolic substrates [63]. In addition, microenvironment-responsive designs, activated by pH, bacterial enzymes, or redox conditions, have improved accuracy in complex infectious niches. Emerging multifunctional platforms further integrate imaging with antimicrobial or phototherapeutic capabilities, highlighting the growing potential of inorganic nanoprobes as sensitive, selective, and versatile tools for infection diagnosis and theranostics. This section will discuss the latest progress of inorganic fluorescence probes for imaging bacterial infections.

##### Upconversion nanoparticles

UCNPs are typically composed of an optically inert host matrix doped with sensitizer ions that absorb incoming photons and activator ions that generate luminescence [104]. Their emission arises from an upconversion process in which 2 or more sequentially absorbed low-energy photons, such as NIR light, are converted into a single higher-energy emission photon. This mechanism, together with their excellent photostability and chemical robustness, sets UCNPs apart from conventional organic dyes and QDs. NIR excitation also reduces tissue autofluorescence and light scattering while providing deeper penetration in biological samples. These advantages make UCNPs particularly well suited for in vivo imaging of bacterial infections [105].

Surface functionalization has emerged as a powerful strategy to endow UCNPs with precise, bacteria-specific imaging capabilities. For instance, Ong et al. [104] achieved specific labeling of *E. coli* through anti-*E. coli* antibody. The constructed antibody–UCN conjugate enables specific recognition and stable binding to bacterial surface antigens, achieving efficient and uniform labeling. This approach not only confers excellent targeting capability to the labeling system but also, by leveraging the unique optical properties of UCNs, which emit visible light under NIR excitation, significantly reduces autofluorescence background in biological samples, thereby yielding imaging results with a high signal-to-noise ratio. The authors compared the photostability of UCNP-labeled bacteria to those labeled with green fluorescent protein, demonstrating that the UCNPs maintained stable luminescent performance under continuous irradiation and significantly outperformed fluorescent proteins, which are readily photobleached [104]. In addition, surface modification to enhance electrostatic adsorption has also been proved to be an effective approach for bacterial capture [106]. Xu et al. [106] modified MAu@Ag@MPBA with positively charged quaternary ammonium salts and produced UCNPs@QAS probes. The UCNPs@QAS were used to detect a range of pathogenic bacteria, including *S. aureus*, *P. aeruginosa*, and *E. coli*.

Notably, the interaction between polymyxin and LPS offers a highly specific mechanism for recognizing Gram-negative bacteria. Polymyxin antibiotics (e.g., polymyxin B) are cationic cyclic lipopeptides. The multi-cationic regions in their structure enable specific binding to LPS components in the outer membrane of Gram-negative bacteria [107]. Based on this mechanism, Qiao et al. [108] covalently attached polymyxin B to the carboxylated surface of NaYF4:Yb,Tm,Fe nanoparticles to construct a highly specific detection probe PMB-UCNPs. This probe effectively detects *E. coli* at concentrations as low as 36 CFU/ml and exhibits low cross-reactivity, even in the presence of *S. aureus* and *Bacillus subtilis*, highlighting its potential as a powerful tool to detecting Gram-negative bacteria in complex environments [108].

##### Metal nanoclusters

MNCs are ultrasmall particles comprising several to hundreds of metal atoms, such as Au, Ag, or Cu [109]. Because their size approaches the Fermi wavelength of electrons, MNCs possess discrete energy levels and a large specific surface area, endowing them with molecule-like characteristics [110]. These unique electronic structures result in outstanding optical and chemical properties, including strong photoluminescence, high photostability, and tunable fluorescence emission [91].

Among various strategies for bacterial imaging using MNC probes, the label-free detection approach based on electrostatic interactions between MNCs and bacterial surfaces is the most widely adopted. For example, Li et al. [111] developed cationic bovine serum albumin protected gold silver nanoclusters (cBSA AuAgNCs), which significantly improved the recognition and real-time imaging ability of bacterial infection through its NIR fluorescence emission and positive surface charge characteristics. Its NIR fluorescence emission (~702 nm) enables deep tissue penetration with minimal biological background, making it suitable for in vivo imaging, while the abundance of surface positive charges promotes strong and specific electrostatic adsorption onto negatively charged bacterial membranes, achieving high-contrast labeling without cellular internalization. Bacterial metabolic pathways also offer unique advantages in pathogen-specific detection. An “on–off–on” type fluorescence probe based on BSA-stabilized gold nanoclusters (AuNCs) was developed by Yan et al. [112] for the rapid and specific detection of *E. coli*. The probe was quenched in the presence of Cu^2+^, but restored by *E. coli* through its specific copper-binding and reduction pathway, enabling rapid and selective detection of the bacterium and subsequent screening of bactericides. This method enables visual detection of *E. coli* at concentrations as low as ~100 CFU/ml within 0.5 h and facilitates rapid pathogen identification and drug susceptibility assessment in simulated contaminated water and sepsis blood samples, demonstrating considerable potential for environmental monitoring and clinical diagnostics (Fig. 4A and B) [112].

**Fig. 4.**
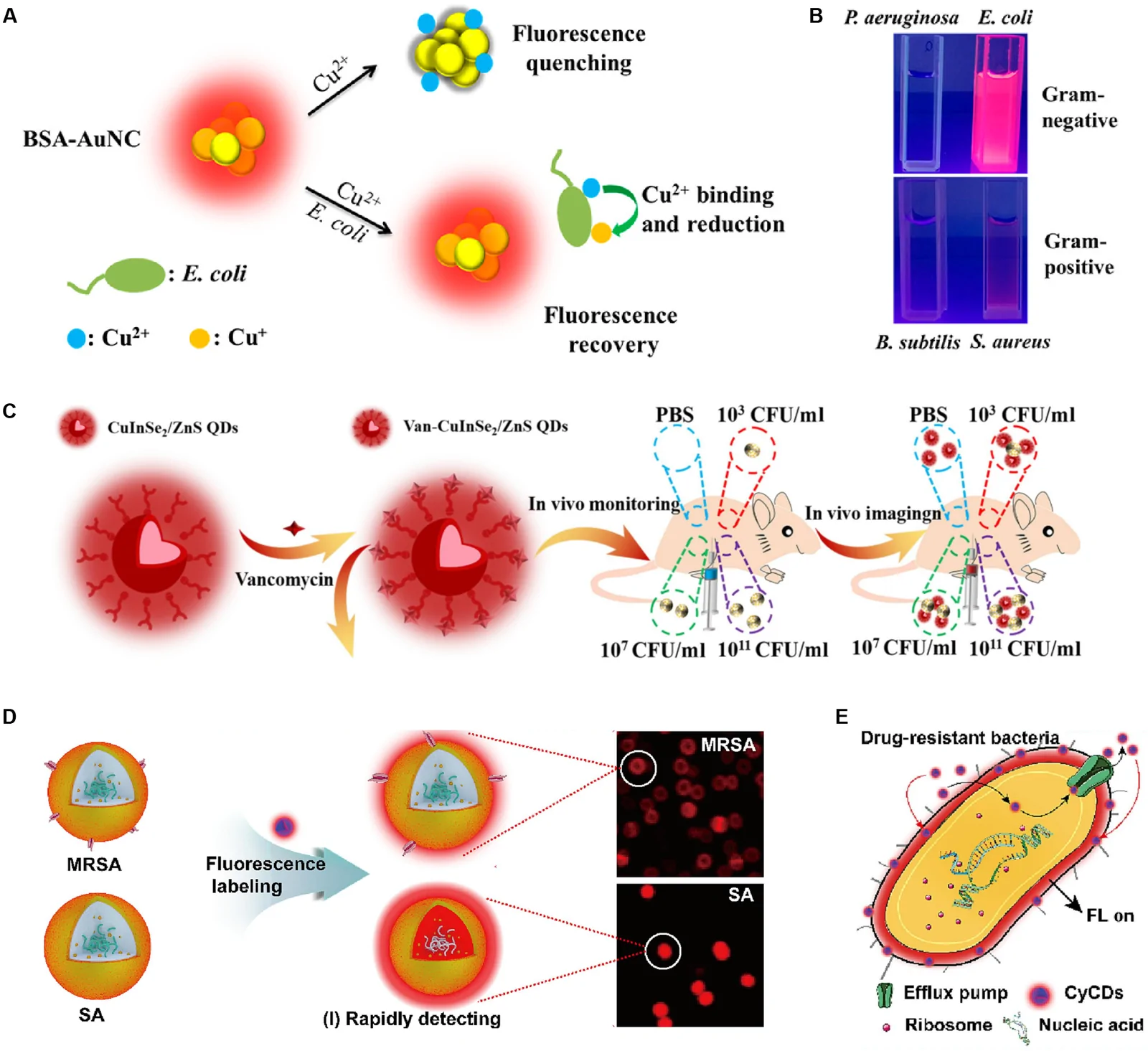
Design strategies and representative applications of inorganic fluorescent nanoprobes for bacterial infection imaging. (A) Schematic illustration of an “on–off–on” fluorescence sensing strategy based on Cu^2+^-mediated quenching and bacteria-triggered recovery of BSA-stabilized gold nanoclusters for rapid *E. coli* detection and bactericide screening [112]. Copyright 2018, American Chemical Society. (B) Representative fluorescence images showing the differential responses of the AuNC-Cu^2+^ system toward various bacterial species after incubation (10^6^ CFU ml^−1^, 0.5 h), highlighting its selectivity [112]. Copyright 2018, American Chemical Society. (C) Schematic representation of vancomycin-modified NIR-II-emissive CuInSe_2_/ZnS QDs for targeted in vivo imaging of *S. aureus* infections [117]. Copyright 2025, Elsevier. (D) Confocal fluorescence imaging illustrating the interaction of CD-based probes with bacterial cells for discrimination and visualization [123]. (E) Proposed imaging mechanism of CyCDs for distinguishing drug-resistant and drug-susceptible bacteria based on differential intracellular distribution patterns [123]. Copyright 2023, Elsevier.

To achieve higher specificity and sensitivity in bacterial detection, integrating multiple recognition elements with diverse signal output modes has emerged as an important research direction. For instance, Cheng et al. [113] developed a dual-recognition strategy for the highly specific detection of *S. aureus* in complex samples. Their design effectively integrates the high specificity of aptamers with the broad-spectrum affinity of antibiotics. Specifically, aptamer-modified magnetic beads are used to capture and enrich the target bacteria, while vancomycin-protected gold nanoclusters (AuNCs@Van) bind to the terminal d-Ala-d-Ala peptides of the bacterial cell wall, thereby providing a fluorescent signal for detection. This approach achieved highly sensitive detection of *S. aureus* with a limit of detection (LOD) of 16 CFU/ml. Notably, it maintained specificity for *S. aureus* at concentrations as low as 70 CFU/ml, even in the presence of a high background of interfering contaminants (up to 10^8^ CFU/ml). The strategy was successfully demonstrated in the analysis of real samples such as milk and serum [113]. Overall, this dual-recognition mode, which combines magnetic separation with fluorescence detection, significantly improves the reliability and accuracy of pathogen detection in complex backgrounds.

##### Quantum dots

QDs are nanoscale semiconductor particles, typically 2 to 10 nm in diameter, that exhibit size-dependent optical and electronic properties due to quantum confinement effects [114]. When excited by light, they can emit bright, tunable fluorescence with narrow emission spectra and high photostability, making them valuable in bioimaging. In 1998, Bruchez et al. [115] first applied QDs as fluorescent nanoprobes for biological staining and diagnosis. Since then, the biomedical applications of QDs have expanded continuously and now widely used in the imaging of bacterial infections.

QDs modified with antibiotics serve as effective fluorescent probes for bacterial detection, combining the high-resolution imaging capabilities of QDs with the cell wall-specific targeting ability of antibiotics. For example, Huang et al. [116] developed an acidic pH/photothermal dual-responsive nanosystem, Ag_2_S@ZIF-Van, composed of Ag_2_S QDs, ZIF-8, and vancomycin. This system enables targeted NIR fluorescence imaging of *S. aureus* infections in murine subcutaneous inflammation and wound infection models while achieving synergistic antibacterial therapy through combined Zn^2+^ release, vancomycin activity, and photothermal effects. Similarly, Geng et al. [117] developed a NIR-II fluorescent nanoprobe based on vancomycin-modified CuInSe_2_/ZnS QDs for dynamic monitoring and photothermal therapy of bacterial infections. The probe specifically targets *S. aureus* and enables real-time fluorescence imaging of the interaction between the bacteria and the host immune system during infection. The study employed both a subcutaneous abscess model and a skin wound infection model in mice to demonstrate the probe’s capability for in vivo tracking and photothermal eradication of *S. aureus* (Fig. 4C).

QDs can also be functionalized with nucleic acids to create versatile fluorescent probes for bacterial detection, leveraging the inherent specificity of nucleic acids. Xiong et al. [118] reported a bivalent DNA-encoded photoactivatable QD killer for sequential cellular penetration, intracellular bacterial imaging, and targeted elimination. By designing a chimeric DNA template containing phosphorothioate (PS) and phosphodiester (PO) regions, the team guided the synthesis of CdTe@CdS@ZnS QDs decorated with an aggregation-induced emission (AIE)-active photosensitizer. These QDs were further conjugated with bivalent DNA aptamers targeting both M2 macrophages and bacteria, resulting in a multifunctional nanoprobe capable of targeted delivery, imaging, and photodynamic bactericidal activity. Experiments demonstrated that the QD killer could enter infected macrophages via receptor-mediated endocytosis and specifically recognize and label intracellular bacteria.

##### Carbon dots

CDs are zero-dimensional carbon-based materials with sizes less than 10 nm. They typically possess an *sp*^2^-hybridized, conjugated carbon core and are functionalized with various organic groups, such as carboxyl (COOH), amino (NH_2_), hydroxyl (OH), carbonyl (C=O), ether (C–O–C), amide (C–N), and thiol (SH), as well as polymer chains [119]. CDs offer several key advantages as fluorescent imaging probes over traditional QDs, including simple and cost-effective synthesis, high chemical stability, and excellent biocompatibility. Their small size, resistance to photobleaching, tolerance to high temperatures and ionic strength, and ability to retain performance after prolonged storage make them particularly well-suited for biological applications [120].

In recent years, the potential of CDs in deep-tissue imaging has attracted increasing attention. Due to their tunable emission spectra, high tissue penetration capability, and good photostability, CDs have become a powerful tool for achieving high-contrast, high signal-to-noise ratio imaging in vivo. For example, Song et al. [121] developed chiral CDs (D-CDs) synthesized from d-cysteine, which enable selective identification and imaging of Gram-positive bacteria and fungi while also providing synergistic photodynamic antibacterial activity.

In bacterial infection imaging, CDs not only are used to locate infections but also have been explored for distinguishing between different bacterial types or even phenotypes. Duan et al. [122] developed NIR carbonized polymer dots (NIR-CPDs) with a core–shell structure, which can specifically recognize *S. aureus* and embed into its cell membrane, enabling high-contrast imaging in both subcutaneous and deep infection models while also exhibiting photodynamic antibacterial functions. Addressing the challenge of identifying drug-resistant bacteria, Liu et al. [123] designed second NIR (NIR-II) emissive CDs (CyCDs). With their positively charged surface, CyCDs rapidly bind to negatively charged bacteria. In susceptible bacteria, the probe evenly labels the cytoplasm, whereas in bacteria with efflux pump systems, CyCDs are confined to the cell wall, outlining the bacterial shape and thereby allowing rapid distinction between drug-resistant and susceptible strains in vitro. In a mouse infection model, changes in the fluorescence signal of this probe reflected bacterial load in real time, providing a visual imaging basis for monitoring infection progression in vivo (Fig. 4D and E).

The integration of imaging capabilities with therapeutic functions represents a growing trend in nanomaterial-based infection management. In addition to the imaging probes discussed above, several recent studies have demonstrated the translational potential of multifunctional nanomaterials in combating bacterial infections. For example, Lei et al. [124] developed a bioactive nanocomposite sponge that achieves simultaneous hemostasis and antimicrobial therapy, addressing 2 major causes of post-traumatic mortality. Lu et al. [125] designed a photo-responsive hollow manganese/carbon hybrid nanosphere that serves as both a nanozyme and a photothermal agent for synergistic disinfection of multidrug-resistant wound infections and promotion of wound healing. These examples underscore the broad therapeutic applicability of nanomaterials beyond diagnostic imaging and illustrate the complementary roles of imaging and therapy in next-generation antibacterial platforms [126].

### Chemiluminescence imaging

Chemiluminescence imaging is a technique that enables sensitive, real-time visualization of biological targets using probes that emit light through a chemical reaction in response to specific analytes or biological events, without requiring external light excitation [127]. This contrasts sharply with fluorescence imaging, which depends on external light excitation and is often hindered by tissue autofluorescence and limited penetration depth. The fundamental principle of chemiluminescence involves a chemical reaction that excites a molecule from its ground state to an electronically excited state, which then releases energy as a photon upon returning to the ground state [128]. In direct chemiluminescence, a reactant or product is directly excited by reaction energy to emit light, whereas in indirect chemiluminescence, or chemiluminescence resonance energy transfer (CRET), a reaction-generated excited intermediate transfers energy nonradiatively to a nearby fluorophore, which then emits light [129]. Chemiluminescence offers significant advantages for bacterial infection imaging, including high sensitivity, minimal background interference, and enhanced tissue penetration, enabling accurate and real-time visualization of pathogens in complex biological environments [130].

The most commonly used chemiluminescent substrate in bacterial infection research is luminol [131]. Upon oxidation at sites of infection or inflammation, luminol can be excited to a high-energy state and emits blue light (~425 nm) as it returns to the ground state. In addition to luminol, several other well-established chemiluminescent substrates have been reported, such as acridinium esters, peroxyacetic acid salts, 1,2-dioxane, and their derivatives [132]. Currently, the research focus in this field has shifted to probe engineering aimed at enhancing imaging specificity and signal-to-noise ratio. For the luminol system, strategies such as designing it as a prodrug activated by bacteria-specific enzymes (e.g., β-lactamase) or encapsulating it within bacteria-targeting nanocarriers are being actively pursued to achieve direct pathogen detection, rather than merely indicating inflammation [133].

Chemiluminescence probes are designed and tailored to meet specific preclinical needs in bacterial infection situations. For deep-tissue infections, Zhou et al. [134] reported a NIR chemiluminescent nanoparticles (ALPBs) composed of luminol, an AIE dye (TTDC), a photothermal polymer (PCPDTBT), and a nitric oxide donor (BNN6) within a poly(lactic-co-glycolic acid)-polyethylene glycol 5000 (PEG5000) matrix. This system achieves chemiluminescence imaging-guided synergistic photothermal–nitric oxide therapy for deep-seated bacterial-infected muscle tissue. For real-time detection, Chen et al. [135] developed a persistent and intense chemiluminescence system using copper nanoprobes functionalized with 4-mercaptophenylboronic acid (MPBA), which exhibits enzyme-like properties. The core material, CuSe nanoparticles, act as a nanoenzyme to catalyze the luminol–H_2_O_2_ reaction, enabling high-precision real-time detection of *S. aureus* and *E. coli* (Fig. 5A). To address specific clinical requirements following kidney transplantation, Yu et al. [136] developed a multifunctional platform based on persistent chemiluminescence for the highly sensitive detection and photothermal inactivation of *E. coli*. This platform employs antibody-functionalized Fe_3_O_4_ nanoparticles for the specific capture and magnetic separation of target bacteria, along with copper–selenium nanoparticles (Cu_2−*x*_Se NPs) that catalyze the luminol–H_2_O_2_ system to produce stable and continuous chemiluminescence signals. This makes it particularly suitable for point-of-care diagnosis and treatment of infections following kidney transplantation.

**Fig. 5.**
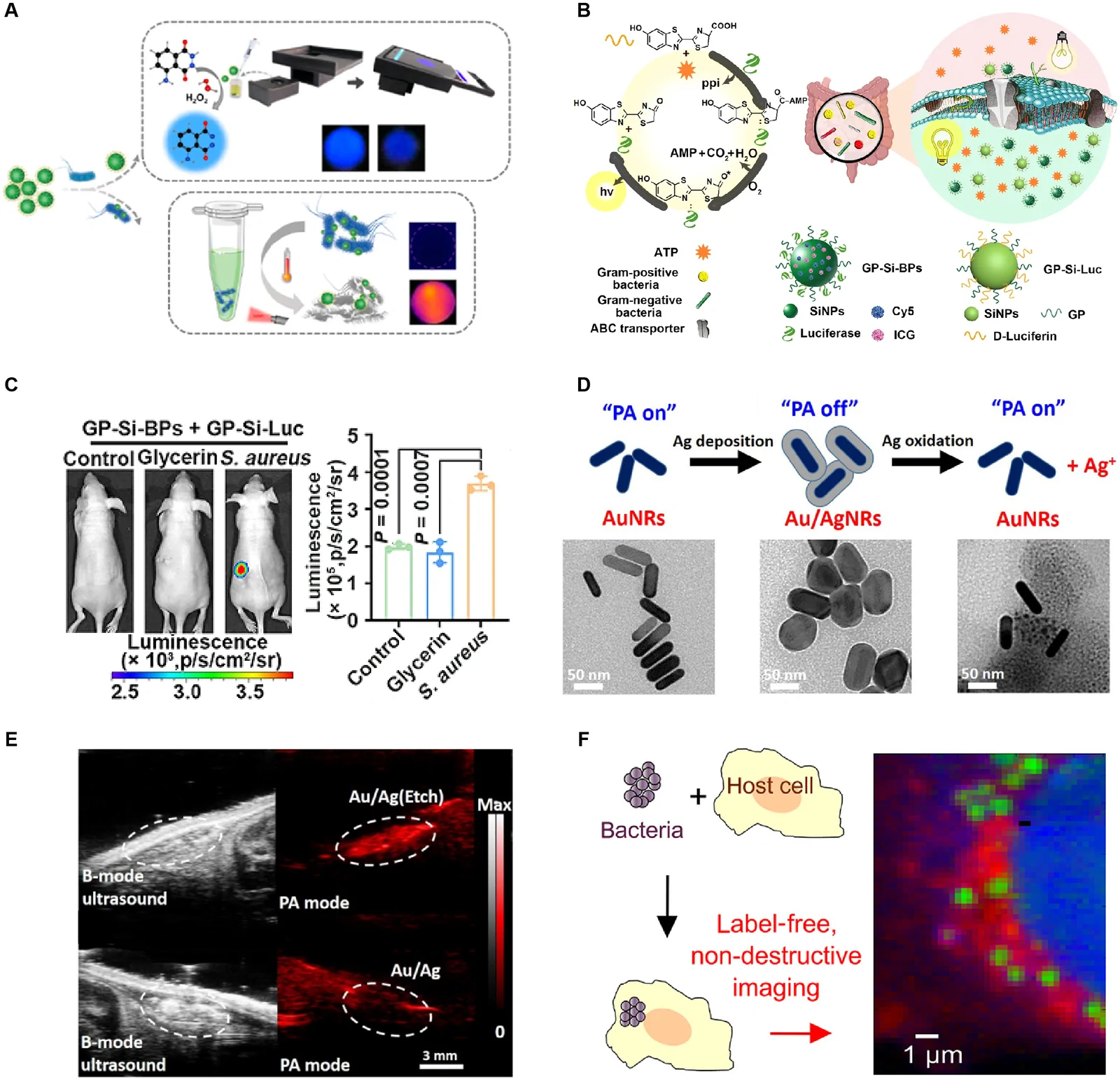
Design strategies and representative applications of nonfluorescence optical nanoprobes for bacterial infection imaging. (A) Comparison of chemiluminescence duration and signal stability between the luminol–H_2_O_2_–CuSeNPs@MPBA system and control formulations, demonstrating enhanced and persistent light emission [135]. Copyright 2022, American Chemical Society. (B) Schematic illustration of a Trojan horse strategy exploiting bacterial ABC sugar transporters for selective delivery of bioluminescent nanoprobes, enabling ATP-dependent in vivo visualization of bacterial infections [141]. Copyright 2023, Springer Nature. (C) Bioluminescence imaging of healthy, glycerin nephritis-bearing, and *S. aureus* nephritis-bearing mice using Trojan BLI probes (mean ± SD, *n* = 3) [141]. Copyright 2023, Springer Nature. (D) Experimental schematic and transmission electron microscopy (TEM) images of Au nanorods, Au/Ag core–shell nanorods, and oxidant-etched Au/Ag nanorods, illustrating infection-triggered photoacoustic signal activation [150]. Copyright 2018, American Chemical Society. (E) PAI of the Au/AgNR system in vivo, with B-mode ultrasound (grayscale) and photoacoustic (red) images showing signal enhancement (730%, *P* < 0.01) after systemic silver etchant administration (intraperitoneally). Scale bar, 3 mm [150]. Copyright 2018, American Chemical Society. (F) Label-free Raman imaging of *S. aureus* residing within infected EA.hy926 endothelial cells, demonstrating high-resolution intracellular bacterial identification based on intrinsic molecular fingerprints [157]. Copyright 2015, American Chemical Society.

### Bioluminescence imaging

Bioluminescence imaging (BLI) operates through enzyme-catalyzed reactions in which bioluminescent are converted into excited intermediates that undergo oxidation and emit visible light. The emitted light can penetrate living tissues, enabling for precise monitoring of subtle changes in physiological and pathological processes both in vitro and in vivo [137]. A typical bioluminescent system comprises a luciferase enzyme, a luciferin substrate, and oxidants, with some systems also requiring ATP (adenosine triphosphate) or NADH [reduced form of nicotinamide adenine dinucleotide (oxidized form)] as an energy source [138]. Similar to chemiluminescence imaging, BLI exhibits low background interference and causes no tissue damage because it does not require external excitation light, allowing for convenient noninvasive imaging. Since its initial discovery and report in fireflies in 1900, the d-luciferin/luciferase system [139] has become one of the most extensively utilized bioluminescent platforms for in vivo imaging, owing to the ease of luciferin synthesis and its outstanding stability and biocompatibility under physiological conditions [140]. These advantages have led to the growing use of BLI for tracking bacterial infections in living organisms with high sensitivity and specificity.

Building on the advantages of bioluminescent systems for bacterial infection imaging, researchers have developed strategies to enhance probe delivery efficiency and signal specificity. Zhang et al. [141] developed a BLI probe based on a “Trojan horse” strategy, which utilizes bacteria-specific ATP-binding cassette (ABC) sugar transporters to deliver glucose polymer (GP)-modified silicon nanoparticles into bacterial cells. In multiple infection models, the probe enabled specific imaging of *S. aureus* kidney infections (~1.0 × 10^8^ CFU) and *Salmonella typhimurium* intestine infection (~1.0 × 10^9^ CFU), with a detection limit of 10^5^ CFU. It also distinguished bacterial from nonbacterial inflammation [e.g., in glycerol- or dextran sulfate sodium (DSS)-induced models]. Additionally, ex vivo imaging of clinical samples successfully identified 10 different pathogenic bacterial species in vitreous humor from patients with endophthalmitis (Fig. 5B and C) [141].

Alternatively, direct labeling of the bacterial cell wall has also been explored for BLI of infection. For example, Song et al. [142] reported a novel metabolic labeling strategy for imaging newly synthesized bacterial peptidoglycan. They designed an aryl azide-modified d-amino acid, which could be incorporated into the newly formed peptidoglycan of bacterial cell walls. Meanwhile, they synthesized a “caged” d-luciferin precursor that specifically reacts with the azide group on peptidoglycan via a Staudinger ligation, releasing free d-luciferin and, consequently, activating luciferase to produce a strong bioluminescent signal. This approach enabled specific bacterial imaging and provided a powerful tool for studying microbial dynamics in vivo.

Furthermore, integrated biosensing platforms offer distinct advantages for the rapid and sensitive detection of bacteria while simultaneously enabling effective disinfection. In this context, Zhou et al. [143] developed a nanozeolite imidazolium framework (ZIF-90@Luc-AMP)-based biocomposite that integrates capture–detection–disinfection functionalities into a single platform. The platform utilizes phage-functionalized materials to specifically capture and enrich target pathogenic bacteria. Subsequently, synergistic action of phages and antimicrobial peptides lyses the bacteria, rapidly releasing intracellular ATP. Finally, luciferase embedded within the composite catalyzes an ATP-dependent bioluminescence reaction, enabling sensitive detection of bacterial loads as low as 20 CFU/ml within 30 min while simultaneously achieving effective disinfection [143].

### Photoacoustic imaging

PAI, first conceptualized by Alexander Graham Bell in 1880 [144], is a nonionizing and noninvasive imaging technique that integrates optical excitation with ultrasonic detection. When pulsed laser irradiation causes endogenous chromophores in biological tissues to absorb light energy, it produces a rapid thermoelastic expansion that generates ultrasonic waves. These photoacoustic signals are subsequently captured by ultrasonic transducers and computationally reconstructed to produce high-contrast images reflecting the tissue’s optical absorption properties [145]. Currently, PAI is widely applied in fundamental studies of biological tissues and in vivo small-animal imaging. To address the limited penetration depth of light in tissue, exogenous contrast agents, including biocompatible nanoparticles, chemical dyes, and reporter genes, have been introduced [146]. The incorporation of these agents not only improves imaging depth in both animal models and human tissues but also greatly expands the capabilities of PAI, enabling highly specific visualization of pathological processes, including bacterial infections.

In bacterial infection imaging, PAI has been advanced through the development of smart inorganic nanomaterial-based probes that accumulate at infection sites and respond to the local microenvironment. For instance, Liu et al. [147] developed a Janus-structured nanomotor composed of gold nanorods, silica, and copper sulfide (AuNR-SiO_2_-Cu_7_S_4_), which enables precise antibacterial therapy guided by dual-region NIR PAI. In a mouse subcutaneous abscess model infected with MRSA, the study demonstrated that the nanomotor selectively accumulated at infection sites, enabling effective noninvasive imaging. Another approach involves designing probes that respond specifically to bacterial metabolites. Yuan et al. [148] developed a bismuth-based metal–organic framework (Bi-MOF) platform that serves as a H_2_S-responsive photoacoustic probe for imaging bacterial infections. The probe operates by reacting with elevated endogenous H_2_S produced by methicillin-tolerant *S. aureus*, forming bismuth sulfide (Bi_2_S_3_) in situ. Under 690-nm laser irradiation, Bi_2_S_3_ generates a strong photoacoustic signal, enabling precise and real-time visualization of infection sites with deep tissue penetration. Similarly, Yang et al. [149] developed an infection microenvironment-responsive diagnostic platform based on copper oxide nanoparticles (Cu_2_O NPs) for NIR-II PAI of subcutaneous abscesses caused by MRSA. The nanoplatform specifically responds to elevated H_2_S and H_2_O_2_ levels at infection sites: Reaction with H_2_S produces Cu_9_S_8_ nanoparticles with strong absorption in the NIR-II window, enabling high-contrast imaging and precise differentiation between infected and healthy tissues.

Additionally, modulating the chemical state of nanomaterials to enable signal switching represents an effective strategy to improve imaging specificity. For example, Kim et al. [150] reported gold/silver hybrid nanoparticles (Au/AgNRs) comprising a gold nanorod core surrounded by a silver shell. Under physiological conditions, the silver shell suppresses the photoacoustic signal. However, at infection sites, oxidants such as ROS induce etching of the silver shell, thereby “unlocking” and markedly enhancing the photoacoustic signal from the gold nanorods in the NIR region. In a murine model of subcutaneous MRSA skin infection, the triggered silver etching resulted in a 730% increase in photoacoustic signal intensity compared to the pre-etching baseline (Fig. 5D and E) [150].

While PAI offers high spatial resolution and contrast for visualizing bacterial infections, several challenges limit its widespread application. Tissue penetration remains restricted, making it difficult to image deep-seated infections such as those in the liver or kidneys [151]. Image quality can also be compromised by acoustic distortions caused by air-filled cavities or bone structures, as well as artifacts arising from limited detection angles or bandwidth. Additionally, the specificity of PAI for bacterial infections depends heavily on the design of targeted contrast agents, which must selectively accumulate at infection sites and respond to the microenvironment. Addressing these challenges through advanced nanoprobes, responsive contrast agents, and multimodal imaging strategies is essential for improving sensitivity, accuracy, and clinical applicability in bacterial infection imaging.

### Raman imaging

Raman imaging is a label-free or probe-assisted optical imaging technique that visualizes the molecular composition of samples based on inelastic scattering of light, known as the Raman effect [152]. As a powerful analytical tool, Raman imaging combines spectral and spatial information to create detailed molecular maps. It collects thousands of Raman spectra from different spatial locations across a sample and then generates pseudo-color images that reflect the sample’s composition and structure based on spectral features such as peak intensity, position, and width [153,154]. For bacterial infection detection, Raman imaging probes employ nano-engineered particles to generate characteristic signals that can directly identify pathogens. The specificity and selectivity of these probes can be further enhanced by functionalizing the nanoparticles with recognition elements such as small molecules, antibodies, or aptamers.

Various Raman imaging probes have been developed for bacterial infections in animal models, integrating targeting, imaging, and therapeutic functions into a single platform. For instance, Gao et al. [155] developed a multifunctional NIR SERS nanoprobe through the self-assembly of a gold nanostar–MoS_2_–hyaluronic acid complex (AMD@HA). The probe demonstrated high-performance SERS imaging capabilities, achieving a detection limit as low as 10^2^ CFU/ml for MRSA and enabling rapid acquisition of Raman images (40 × 40 pixels in 120 s) with high specificity between infected and healthy tissues. In MRSA-infected wound and subcutaneous abscess mouse models, AMD@HA not only provided real-time, sensitive visualization of bacterial colonization but also exhibited excellent biocompatibility and targeted accumulation at infection sites, supporting its potential for image-guided antibacterial therapy. Beyond these basic functions, improvements in detection sensitivity and the extension of monitoring duration have emerged as key goals for optimizing probe performance. He et al. [156] developed an integrated theranostic platform based on gold–silver nanoshells (AuAgNSs). By conjugating the Raman reporter 3,3ʹ-diethylthiatricarbocyanine iodide (DTTC) to the nanoshells, the system enables sensitive SERS imaging, achieving a detection limit as low as 300 CFU/ml for MRSA. This high sensitivity permits prolonged, real-time monitoring of bacterial infection progression and treatment response in vivo, with continuous tracking capability extending over 8 d.

Frontier research has focused on overcoming the core limitations of SERS technology, including interference from endogenous tissue fluorescence and the challenges of precise probe positioning in complex biological environments. Große et al. [157] established a label-free Raman imaging platform that enables high-resolution, 3-dimensional localization of *S. aureus* within living endothelial cells. By employing confocal Raman microscopy coupled with multivariate spectral analysis (N-FINDR algorithm), their approach generates detailed pseudo-color maps that distinguish intracellular bacteria from host cellular components based on intrinsic biochemical signatures, without the need for exogenous labels (Fig. 5F). Zhou and Li [158] addressed the core challenges in SERS-based bioimaging, such as interference from endogenous tissue fluorescence and insufficient targeting in complex biological environments, by developing a short-wave infrared (SWIR) resonant Raman reporter through an ionic molecular engineering strategy. By converting neutral metal dithiolene compounds into ionic dark quenchers, they constructed surface-enhanced resonance Raman scattering (SERRS) nanoprobes that exhibit near-zero fluorescence background. These nanoprobes demonstrated remarkable performance metrics: an SBR enhanced by 18-fold, an ultralow detection limit of 0.81 pM, a photothermal conversion efficiency of 61.4%, and exceptional photostability with a photobleaching time constant of 10,155 s. In vivo, the functionalized nanoprobes enabled high-specificity SERRS imaging at single-cell resolution and real-time therapeutic monitoring in both multidrug-resistant *Acinetobacter baumannii* biofilm-infected skin wounds and MRSA-induced keratitis models (Table 1).

**Table 1.** Representative optical nanoprobes for bacterial infection diagnosis

Probe type	Targeting strategy	Nanoprobes	Pathogens	Detecting sensitivity	Infection model	Ref.
Organic fluorescent probe	Cell wall	BacGO	Gram-positive bacteria (e.g., *S. aureus*)	10^5^ CFU/ml	Corneal infection	[82]
	Metabolic pathway targeting (d-alanine)	HF-D-Ala NPs	Metabolically active bacteria	–	–	[83]
	Phage targeting + AIE	MS2-DNA-AIEgen	Intracellular *E. coli*	84.5 pM DNA	Diabetic wound infection	[84]
	Enzyme-responsive (nitroreductase)	Cu_2–_Se@BSA@NTRP	*E. coli*, *S. aureus*	21.19 ng/ml NTR	Bacterial keratitis	[97]
	Enzyme-responsive (β-lactamase)	BIN-3	β-Lactamase-expressing bacteria	2 × 10^6^ CFU	Subcutaneous abscess model, myositis	[98]
	NTR + PBP	Nap-CefTTPy	*S. aureus* and other pathogens	0.0453 μg/ml NTR	Wound infection	[100]
	Charge-based (bacterial membrane)	BacVue	Uropathogenic *E. coli* (UPEC)	–	Urinary tract infection (UTI)	[88]
	PABA	PABA-DCM	Live bacterial infection sites	–	Wound infection	[92]
Inorganic fluorescent probe (UCNPs)	Antibody-based targeting	Antibody–UCN conjugate	*E. coli*	–	–	[104]
	Charge adsorption	UCNPs@QAS	*S. aureus*, *P. aeruginosa*, *E. coli*	20 CFU/ml (buffer)	–	[106]
	Antibiotic-based (polymyxin B)	PMB-UCNPs	Gram-negative bacteria	36 CFU/ml (PBS)	–	[108]
Inorganic fluorescent probe (MNCs)	Electrostatic adsorption	cBSA AuAgNCs	Bacterial membrane	89 CFU/ml	–	[111]
	Metabolic response (copper binding)	BSA-AuNCs	*E. coli*	~100 CFU/ml	–	[112]
	Aptamer + antibiotic	Aptamer-MB + AuNCs@Van	*S. aureus*	16 CFU/ml (analytical assay; milk/serum validation)	–	[113]
Inorganic fluorescent probe (QDs)	Vancomycin	Ag_2_S@ZIF-Van	*S. aureus*	1 × 10^6^ CFU/ml	Subcutaneous inflammation, full-thickness skin wound infection	[116]
	Vancomycin	CuInSe_2_/ZnS-Van QDs	*S. aureus*	–	Subcutaneous abscess model, skin wound infection	[117]
	Bivalent aptamer targeting	CdTe@CdS@ZnS-aptamer	Intracellular bacteria	–	Wound infection	[118]
Inorganic fluorescent probe (CDs)	Chiral recognition	D-CDs	Gram-positive bacteria/fungi	–	Full-thickness skin wound	[121]
	Membrane embedding	NIR-CPDs	*S. aureus*	–	Subcutaneous infection	[122]
	Charge recognition + efflux pump differentiation	CyCDs	Drug-resistant vs. susceptible bacteria	–	Subcutaneous infection	[123]
Chemiluminescence probe	Nanozyme-catalyzed	ALPBs	Deep tissue infection	–	Bacterial myositis	[134]
	Nanozyme-catalyzed	CuSe@MPBA	*S. aureus*, *E. coli*	1.25 CFU/ml	–	[135]
	Antibody capture + nanozyme	Fe_3_O_4_ + Cu_2–*x*_Se NPs	*E. coli*	1 CFU/ml	–	[136]
Bioluminescence probe	Sugar transporter-mediated	GP-Si-BPs + GP-Si-Luc	*S. aureus*, *S. typhimurium*	10^5^ CFU	Kidney infection, intestinal infection	[141]
	Metabolic labeling (d-amino acid)	Aryl azide-d-amino acid	Newly synthesized peptidoglycan	–	–	[142]
	Phage capture + ATP detection	ZIF-90@Luc-AMP	Pathogenic bacteria	20 CFU/ml (spiked serum)	–	[143]
Photoacoustic probe	Light-guided	AuNR-SiO_2_-Cu_7_S_4_	MRSA	–	Subcutaneous abscess	[147]
	H_2_S-responsive	Bi-MOF	Methicillin-tolerant *S. aureus*	–	Subcutaneous infection	[148]
	H_2_S/H_2_O_2_-responsive	Cu_2_O NPs	MRSA	–	Subcutaneous abscess	[149]
	Oxidation-responsive (Ag shell etching)	Au/AgNRs	MRSA	–	Subcutaneous MRSA skin infection	[150]
Raman probe	Ligand-mediated (hyaluronic acid)	AMD@HA	MRSA	10^2^ CFU/ml	MRSA-infected wound and subcutaneous abscess	[155]
	Ag^+^ release	AuAgNSs-DTTC	MRSA	300 CFU/ml	Wound infection	[156]

### Comparative advantages and limitations of imaging modalities

Although each optical imaging modality provides unique advantages, their diagnostic performance is determined by a trade-off among sensitivity, specificity, imaging depth, temporal resolution, and clinical practicality (Table 2). Therefore, these modalities should be considered complementary rather than competing approaches, with modality selection depending on the specific diagnostic requirements, including bacterial targets, infection location, and sample complexity.

**Table 2.** Comparative advantages, limitations, and potential applications of optical imaging modalities for bacterial infection diagnosis

Imaging modality	Major advantages	Key limitations	Representative evidence	Ref.
Fluorescence	High sensitivity; structural tunability; multiplexing	Autofluorescence; photobleaching; shallow penetration	BSA-AuNCs detected *E. coli* at ~100 CFU/ml within 0.5 h	[112]
Chemiluminescence	Low background; high sensitivity; high contrast	Substrate dependence; short signal duration; reaction instability	CuSe@MPBA achieved a detection limit of 1.25 CFU/ml for *S. aureus* and *E. coli*	[135]
Bioluminescence	Very low background; longitudinal imaging; high specificity	Reporter/substrate dependence; weak signal; limited penetration	Trojan BLI achieved a 4.05-fold higher SNR than GP-Ce6-SiNP fluorescence imaging in an *S. typhimurium* colitis model	[141]
Photoacoustic	Great imaging depth; high spatial resolution; low background	Optical attenuation; acoustic artifacts; contrast-agent dependence	Cy7-1-maltotriose enabled PA imaging of *E. coli* myositis, with PAI offering reported imaging depths of up to ~4 cm	[197]
Raman/SERS	Molecular fingerprinting; chemical specificity; label-free identification	Weak intrinsic signal; complex instrumentation; SERS variability	Targeted SERS identified 14 bacterial species with 96.1% classification accuracy based on their spectral signatures	[198]

Beyond optical imaging, positron emission tomography (PET) and single-photon emission computed tomography (SPECT) are well suited for deep-tissue and whole-body imaging but involve ionizing radiation and relatively high costs. PET offers high molecular sensitivity and quantitative imaging; for example, ^18^F-fluorodeoxysorbitol (FDS) PET detected *E. coli* burdens of approximately 10^6^ CFU in a murine infection model and has shown clinical potential for Enterobacterales imaging [159]. MRI provides excellent soft-tissue contrast and anatomical resolution without ionizing radiation but has lower molecular sensitivity; bacteria-targeted MRI probes have nevertheless detected *S. aureus* burdens as low as 10^4^ CFU in vivo [160].

Optical imaging therefore occupies a complementary clinical niche. Its high sensitivity, rapid or real-time signal acquisition, flexible bacteria-responsive probe design, and compatibility with compact and relatively low-cost instrumentation are particularly advantageous for superficial and wound infections, endoscopic or intraoperative imaging, and ex vivo or point-of-care diagnostics [27,28]. However, tissue scattering and absorption fundamentally limit imaging depth and whole-body quantification. Thus, whereas PET/SPECT are better suited to deep-tissue and whole-body localization and MRI provides high-resolution anatomical assessment, the near-term translation of optical nanoprobes is likely to focus on applications in which accessibility and rapid molecular readout outweigh the need for deep-tissue penetration [161,162].

## Optical Nanoprobes in the Application of In Vitro Infection Detection

In vitro bacterial infection detection refers to the identification, quantification, or characterization of bacteria and their associated infection biomarkers outside a living organism to evaluate the presence, type, and activity of pathogenic bacteria. It is typically carried out in controlled laboratory settings using clinical samples (e.g., blood, urine, sputum, and wound exudate) or cultured cells. Common in vitro bacterial detection methods include culture, immunological assays, PCR, and other molecular methods [163]. Bacterial culture, which relies on growing organisms on agar media, remains the gold standard for its accuracy and sensitivity, as well as its broad applicability; however, its long turnaround time makes it unsuitable for rapid clinical diagnostics [164]. Enzyme-linked immunosorbent assay (ELISA), an immunological assay based on antigen–antibody recognition, is faster (2 to 4 h), high-throughput, and highly specific, but it generally suffers from lower sensitivity and a higher risk of cross-reactivity or contamination [165]. PCR, a nucleic acid amplification method, provides high sensitivity, high throughput, and a relatively rapid testing time (2 to 4 h), yet requires complex DNA extraction procedures, dedicated instrumentation, and labor-intensive workflows, posing practical challenges for routine laboratory use [166]. In clinical practice, rapid bacterial detection is essential for early diagnosis and precision treatment, allowing clinicians to make timely and appropriate therapeutic decisions, minimize unnecessary antibiotic use, and reduce the risk of pathogen transmission [167].

Recent advances in optical nanoprobes have greatly enhanced in vitro bacterial detection by enabling faster, more sensitive, and more specific identification of pathogens. The nanoprobes are constructed based on nanomaterials that are engineered with tailored optical properties and functionalized with targeting ligands, including antibodies, aptamers, antibiotics, and nucleic acids, to achieve precise pathogen recognition [168]. These nanoprobes offer ultralow detection limits, rapid signal generation, and compatibility with multiplexed or high-throughput assays, addressing key limitations of traditional bacterial detection methods. In the following section, recent development of optical nanoprobes for bacterial detection in urine, blood, and other clinical samples is reviewed.

### Optical detection of urine samples

UTI is an infection that occurs anywhere along the urinary system, including the urethra, bladder, ureters, or kidneys [169]. It is most commonly caused by the invasion and colonization of pathogenic microorganisms within the urinary tract and is considered one of the most prevalent types of healthcare-associated infections [170]. A wide range of pathogens originating from the fecal microbiota, including bacteria, fungi, viruses, and parasites, can contribute to the development of UTIs [171]. Among these pathogens, Gram-negative bacteria, especially UPEC, represent the predominant causative agents [170]. Detection of bacteria in urine is widely regarded as the gold standard approach for diagnosing UTIs. Therefore, there is an urgent clinical need for a simple, rapid, and accurate method capable of directly identifying UPEC-induced infections.

Recent advances in nanoscience have enabled the development of innovative strategies for detecting bacterial pathogens in urine, offering new diagnostic approaches for UTIs [172]. For example, Xin et al. [173] developed a NIR fluorescent probe (NO-AH) that is sequentially activated by the *E. coli* outer membrane protease T (OmpT) and periplasmic aminopeptidase N (APN). The probe consists of a hemicyanine NIR fluorophore linked via a self-immolative PABA spacer to an OmpT-cleavable Arg–Arg peptide. Upon contact with UPEC, OmpT first cleaves the peptide, generating an intermediate that is further processed by APN to release the active fluorophore, producing a ~3-fold fluorescence turn-on at 720 nm. In in vitro bacterial-response assays, the probe achieves a reported analytical sensitivity of ~10^4^ CFU/ml and enables real-time visualization of UTIs in mouse models following intravesical administration. Sun et al. [174] developed a novel electrochemiluminescence biosensor, constructed by modifying an NH_2_-MIL-53(Al) interface with a ruthenium complex labeled with concanavalin A (Ru-Con A). This biosensor enables quantitative detection of the binding between lectins and LPSs of Gram-negative bacteria in urine samples. Bahari et al. [175] developed a ratiometric Förster resonance energy transfer (FRET) aptamer sensor using CDs to quantify *A. baumannii* in urine via fluorescence intensity ratios. The sensor showed a linear range of 2 × 10^3^ to 4.5 × 10^7^ CFU/ml, a detection limit of 3 × 10^2^ CFU/ml, and 98% specificity and provided results within 30 min.

In addition to enhancing detection accuracy, efforts are also focused on improving overall diagnostic efficacy. Researchers frequently integrate pattern recognition algorithms with nanoprobe-based sensor arrays to enable rapid, high-throughput discrimination of diverse bacterial pathogens. Jiang et al. [176] developed an AIE molecule, TPEPYE, based on tetraphenylethylene (TPE), for fluorescent detection of bacterial LPS in artificial urine samples, achieving nanomolar sensitivity. The probe’s positively charged pyridinium group interacts electrostatically with the negatively charged LPS, inducing rapid aggregation on the bacterial surface and a 22-fold fluorescence enhancement, with a detection limit of 370 pM. TPEPYE demonstrates excellent selectivity and sensitivity in high-ionic-strength urine and can effectively differentiate Gram-negative from Gram-positive bacteria, highlighting its potential for clinical monitoring of UTI.

Han et al. [177] further advanced this strategy by developing a robust sensor array. They constructed an electrostatic complex of polyphenyleneethynylene (PPE) and antimicrobial peptides (AMP), combining bacterial-triggered fluorescence release from PPE with AIE from AMP. Using linear discriminant analysis (LDA) and other machine learning methods, their platform accurately identified 20 common urinary tract pathogens, including mixed infections, directly in urine without pre-culture. The system can deliver results within 30 s to 1 h with detection limit of ~10^3^ CFU/ml and accuracy of 96.4% in 50 clinical urine samples. Moreover, it costs roughly one-third of quantitative PCR (qPCR) and shows potential for point-of-care diagnostics and catheter infection monitoring. A dual-fluorescence “on” sensor array developed by Yu et al. [178] shows the ability to identify 20 common urinary tract pathogens and mixed infections within 30 s, with similar detection limit and accuracy. Collectively, these studies highlight the significant potential of integrating optical nanoprobes with artificial intelligence (AI) for the diagnosis of complex infections.

To facilitate the clinical translation of optical nanoprobes, current efforts focus on converting high-performance laboratory assays into portable, multifunctional platforms for real-time infection detection. Zhang et al. [179] integrated fluorescence imaging with paper-based microfluidics, portable optical systems, and smartphones to create a point-of-care platform that can rapidly screen UTI-related bacteria in 10 min to 2 h. Wu et al. [180] developed a long-lasting chemiluminescence/fluorescence dual-mode platform utilizing AIE nanoenzymes for immediate pathogen detection. These AIE nanoenzymes combine oxidase activity with an AIE effect, enabling them to induce visible luminol chemiluminescence. The platform achieved a detection limit of 10^2^ CFU/ml and, without complex sample preparation, detected *E. coli* and *K. pneumoniae* in urine samples within 10 min (Fig. 6A and B) [180]. Chen et al. [135] further advanced this concept by developing a long-lasting chemiluminescence POCT platform using CuSe@MPBA-modified copper nanoprobes. This platform combines portable visual detection with in situ bacterial inactivation. It utilizes the peroxidase-like activity of CuSe@MPBA to catalyze the luminol–H_2_O_2_ system, generating long-lasting luminescence for over 2 h. Incorporating a microfluidic chip and smartphone imaging module allows quantitative analysis within 15 min and enables on-site sample processing to prevent contamination.

**Fig. 6.**
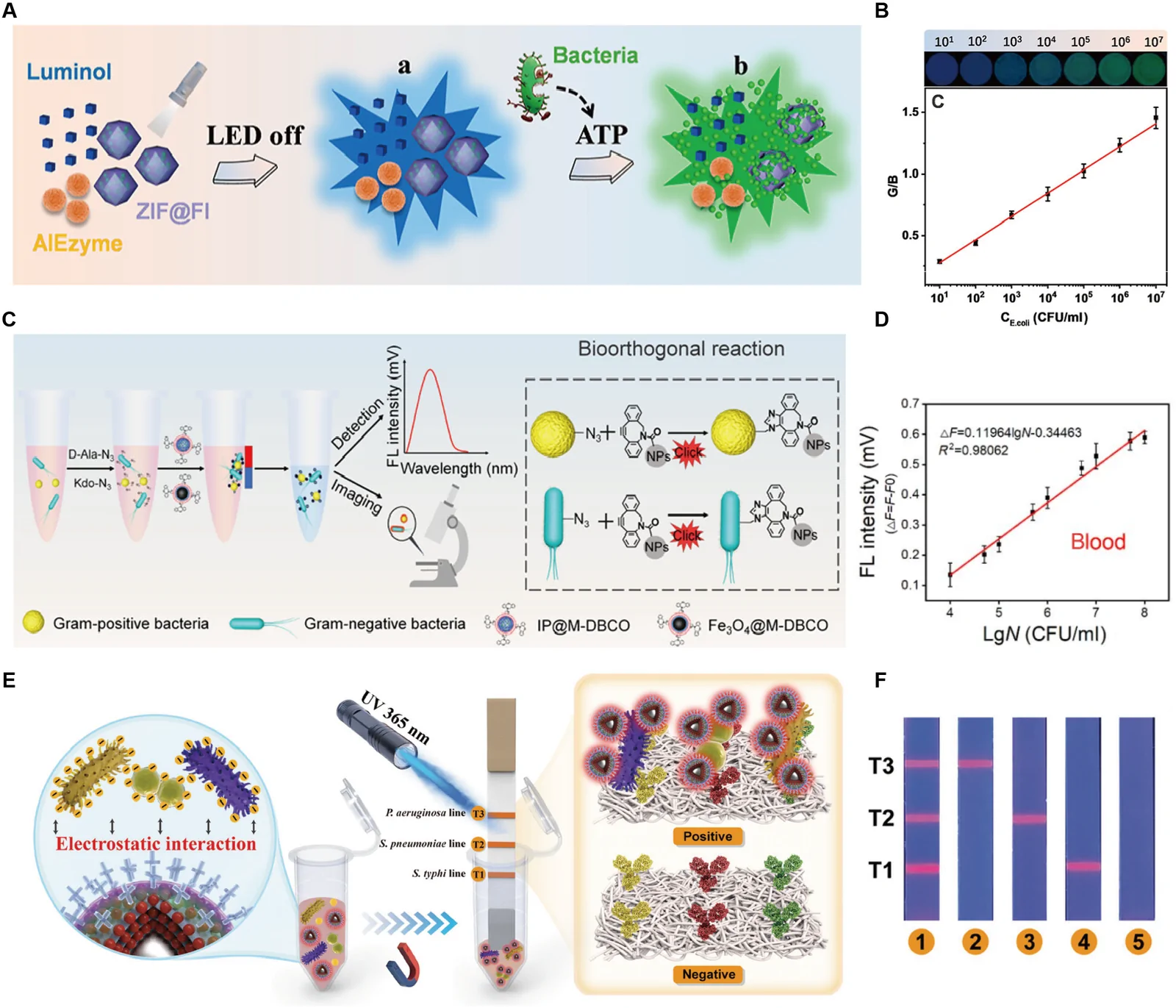
Rapid detection of bacterial infections in representative clinical samples using optical nanoprobes. (A) Schematic illustration of an ATP-triggered CRET-based sensing platform for rapid bacterial detection [180]. Copyright 2023, American Chemical Society. (B) Representative optical images of the sensing system in response to increasing bacterial concentrations (10^1^ to 10^7^ CFU ml^−1^) and corresponding linear correlation between the G/B ratio and *E. coli* concentration (log scale; *n* = 3) [180]. Copyright 2023, American Chemical Society. (C) Schematic representation of a bioorthogonal detection strategy in which metabolically azide-labeled bacteria are selectively captured and visualized using DBCO-modified magnetic NIR nanoprobes [186]. Copyright 2023, American Chemical Society. (D) Linear correlation between fluorescence intensity and bacterial concentration in blood samples, validating the quantitative performance of bioorthogonal nanoprobe-based bacterial capture and identification [186]. Copyright 2023, American Chemical Society. (E) Schematic illustration of multiplexed fluorescent immunochromatographic detection using electropositive MagMQD@Si probes for broad-spectrum bacterial analysis [190]. Copyright 2025, Wiley. (F) Representative fluorescence images of test strips for the detection of mixed bacterial samples, individual target pathogens (*P. aeruginosa*, *S. pneumoniae*, *S. typhimurium*), and a blank control [190]. Copyright 2025, Wiley.

### Optical detection of blood samples

Bacterial bloodstream infections (BSIs) are systemic infections in which bacteria enter and multiply in the bloodstream, potentially leading to sepsis, septic shock, and multi-organ failure [181]. These life-threatening conditions account for approximately one-third of global mortality [182]. BSIs can originate from various sources, including UTIs, respiratory infections, or medical device-associated infections. The most common causative pathogens, including *S. aureus*, *E. coli*, and *P. aeruginosa*, are difficult to detect directly in untreated blood due to their low infection dose (as low as ~100 CFU/ml) amid a background of tens of thousands of blood cells and other interfering substances [183]. Although blood culture remains the clinical gold standard for diagnosing BSI, it is time-consuming and can take several days to yield results [184]. Early and accurate detection of BSIs is therefore critical, as prompt diagnosis and targeted antibiotic therapy significantly reduce morbidity and mortality.

Recent advances in optical nanoprobes are enabling earlier, faster, and more accurate detection of bacterial infections in blood. Li et al. [185] developed a polymyxin B–fluorescein optical nanoprobe (PmBF) that specifically targets LPS on outer membrane vesicles (OMVs) released by Gram-negative bacteria. When combined with nano-flow cytometry, this approach enables quantitative detection of PmBF^+^ extracellular vesicles (EVs) in clinical serum. Infected patients showed significantly higher PmBF^+^ EV levels (~7.90 to 9.61%) than healthy controls (~4.00%). In a murine pneumonia model, PmBF^+^ EVs were detectable as early as 6 h post-infection, well before positive blood cultures, likely due to early OMV release during bacterial colonization and host interaction. Clinical validation further demonstrated that PmBF^+^ EVs effectively differentiate bacterial infections from viral or mycoplasma etiologies, highlighting their potential as a rapid and specific biomarker for early BSI [185].

Following the detection of early infection signals, the causative pathogen needs to be rapidly and accurately identified. To address this need, Zhao et al. [186] developed a NIR fluorescence nanoprobe system based on bioorthogonal reactions. In this approach, bacteria are metabolically labeled with azide groups incorporated into their cell walls, which subsequently serve as universal chemical handles for click reaction with dibenzocyclooctyne (DBCO)-functionalized magnetic NIR nanoprobes. This strategy enables broad-spectrum capture and identification of over 12 common sepsis-causing pathogens, as the metabolic labeling is applicable to both Gram-positive and Gram-negative bacteria through specific integration into peptidoglycan or LPS, respectively. In whole-blood assays, the method achieves a detection limit of 10^4^ CFU/ml within 2.5 h, reduces autofluorescence by operating in the NIR-II window, and demonstrates 97.1% diagnostic sensitivity in 39 clinical sepsis samples (Fig. 6C and D) [186]. Liu et al. [187] developed an optical nanosensing and imaging system based on a ROS-responsive fluorescent probe. Its imaging principle relies on the use of 4 commercially available nanomaterials (Co_3_O_4_, Fe_3_O_4_, single-walled carbon nanotubes, and graphene oxide) to induce differential alterations in intracellular ROS levels upon interaction with bacteria. These ROS signals are then converted into quantifiable fluorescence signals (excitation/emission wavelengths: 488/525 nm) via the universal fluorescent probe 2ʹ,7ʹ-dichlorodihydrofluorescein diacetate (DCFH-DA). The system demonstrates excellent imaging performance, accurately distinguishing 12 bacterial species and 10 mixed bacterial communities, with clear discrimination between Gram-positive and Gram-negative bacteria. In terms of sensitivity, it exhibits a good linear response (*R*^2^ > 0.98) to *S. aureus* within an OD_600_ (optical density at 600 nm) range of 0.005 to 0.2 while maintaining high identification accuracy in complex biological samples such as urine and plasma.

### Optical detection of other samples

Sputum is an important reservoir of bacteria and other microorganisms in the human body. This mucus-based secretion from the lower respiratory tract contains respiratory epithelial cells, bacteria, and various other microbes [188]. When patients present with symptoms such as cough, expectoration, and fever, analysis of sputum samples can aid in identifying bacterial infections, including *S. pneumoniae*, *S. aureus*, and *E. coli* [189]. Precise detection and analysis of bacteria in sputum samples are essential for the timely diagnosis and effective management of respiratory infections.

The advance of optical nanoprobes enables rapid detection of bacteria in sputum samples. For example, Li et al. [190] developed a positively charged magnetic fluorescent nanoprobe (MagMQD@Si^+^) that integrates magnetic enrichment, strong fluorescence output, and broad-spectrum bacterial capture. By directly binding to negatively charged bacteria through electrostatic interactions, this probe enables the detection of pathogens such as *P. aeruginosa*, *S. pneumoniae*, and *Salmonella enteritidis* in sputum samples. Clinical validation showed that the results were highly consistent with qPCR, and the entire detection process required only 15 min, offering an innovative tool for rapid and accurate diagnosis of bacterial infections (Fig. 6E and F) [190]. In the analytical assay, the reported LODs were 8, 31, and 40 cells ml^−1^ for *P. aeruginosa*, *S. pneumoniae*, and *S. typhimurium*, respectively, with a 15-min turnaround, whereas clinical validation was performed separately using 40 authentic sputum samples; these analytical and clinical performance metrics should therefore be interpreted in their respective matrices [190].

In addition, optical nanoprobes have also been optimized with enhanced specificity. For example, Sun et al. [191] validated the dual-recognition platform for detecting target bacteria in authentic human saliva and other clinically relevant samples. The detection was performed by first enriching bacteria from 10-fold diluted saliva samples using aptamer-coated magnetic beads (Apt-MBs), followed by labeling with the broad-spectrum fluorescent probe FcMBL@AuNCs. For spiked MRSA and *K. pneumoniae* (KPC-2 KP) in saliva, the method achieved recoveries of 95.0% to 110.0% with relative standard deviations (RSDs) below 3.0%. The assay maintained a low detection limit of 20 CFU/ml and a wide linear range of 20 to 10^8^ CFU/ml in this complex matrix, demonstrating its robustness and potential for rapid, sensitive diagnosis of bacterial infections in noninvasive clinical specimens [191]. The complete capture-and-fluorescence detection procedure could be completed in approximately 15 min [191]. Thus, the 20 CFU/ml LOD reported for spiked/diluted saliva represents analytical performance in a complex biological matrix rather than prospective clinical sensitivity in an unselected patient cohort.

Besides, bacterial infections are major contributors to dental diseases such as tooth decay, periodontitis, and peri-implantitis [192]. Saliva contains a diverse community of bacteria originating from different oral surfaces, making salivary bacterial detection an emerging and increasingly important research focus. Zhang et al. [193] developed an advanced “electronic tongue” using a DNA-encoded nanoenzyme sensor array. In this system, single-stranded DNA is conjugated to iron oxide nanoenzymes to create programmable sensing units. The nanoenzymes catalyze the substrate 3,3ʹ,5,5ʹ-tetramethylbenzidine (TMB) via their peroxidase-like activity, producing a measurable colorimetric signal. When oral bacteria bind specifically to the DNA, this interaction inhibits the enzymatic activity and generates distinct color patterns. Combined with LDA, the platform produces a unique “fingerprint map” that enables 100% accurate identification of 11 oral bacterial species and 6 proteins. In addition, the nanoenzymes can simultaneously kill cariogenic bacteria by disrupting their membranes and generating ROS. Together, this strategy offers a promising platform for early oral disease diagnosis, personalized treatment, and convenient at-home oral health monitoring [193].

Taken together, the representative platforms discussed above span assay times from seconds to several hours, reflecting substantial differences in target-recognition mechanisms, sample preparation, enrichment requirements, and signal-generation strategies [169]. Although ultralow LODs remain important, rapid clinical diagnosis requires a balance between analytical sensitivity and time-to-result, particularly when bacterial enrichment, metabolic labeling, or multistep washing procedures are required. Therefore, assay duration should be interpreted together with sample matrix, preprocessing requirements, detection sensitivity, and the level of clinical validation when assessing the translational potential of optical nanoprobe-based diagnostics.

## Summary and Perspective

Fluorescent probes remain one of the most versatile and widely used classes of optical nanoprobes due to their highly tunable design. Beyond fluorescence, several other optical modalities provide distinct advantages for infection diagnostics: Chemiluminescence and bioluminescence probes enable ultrasensitive imaging without external excitation, minimizing background noise; photoacoustic probes couple optical contrast with ultrasonic penetration to allow deeper tissue imaging; Raman probes leverage unique spectral fingerprints to distinguish bacterial species with high specificity. Together, these complementary technologies highlight the diverse and powerful toolkit of optical nanoprobes for advancing infection detection and clinical diagnostics.

In clinical settings, optical nanoprobes have already demonstrated strong performance across a variety of specimen types, including blood, urine, and sputum, showcasing clear advantages over traditional diagnostic methods. They enable earlier detection of BSIs than blood culture, allow rapid identification and classification of urinary tract pathogens with clinical validation in patient samples, and support high-throughput screening of respiratory pathogens directly in sputum. Importantly, multiple nanoprobe-based platforms have shown excellent agreement with clinical standards such as qPCR and have been successfully applied to clinic samples, underscoring their translational potential. Additionally, the integration of optical nanoprobes with microfluidics and smartphone-based imaging is speeding the development of portable, real-time diagnostic tools suitable for point-of-care use.

Despite these promising advances and the successful use of optical nanoprobes in detecting pathogens across a variety of clinical specimens, translating these technologies into in vivo diagnosis of bacterial infections remains a significant challenge. Although optical imaging has emerged as a powerful molecular imaging modality in preclinical studies, enabling visualization of bacterial infections in animal models and supporting therapeutic decision-making, its clinical application in humans is still limited. Reliable in vivo bacterial detection faces several intertwined technical and translational challenges. Optical light scattering, absorption, and tissue autofluorescence severely limit imaging depth and signal-to-noise ratio, even within the NIR window, making single-cell detection or low-burden infections in deep organs difficult to resolve [29]. The low pathogen abundance in vivo, often only ~10^2^ to 10^4^ CFU/ml in blood or deep tissues, imposes stringent sensitivity requirements, necessitating strategies such as signal amplification, target enrichment, or highly selective nanoprobe design [162]. In addition, nanoprobe delivery, biodistribution, and clearance present critical practical hurdles, as many inorganic nanoprobes accumulate in the reticuloendothelial system or exhibit slow clearance, complicating repeated dosing and safety evaluation. Potential toxicity and long-term biocompatibility further underscore the need for careful optimization of surface chemistry, degradability, and overall in vivo behavior of nanoprobes. Taken together, current evidence positions optical nanoprobes as promising complements to conventional diagnostic imaging, particularly for rapid infection detection and image-guided intervention. Nevertheless, most studies remain preclinical, and well-designed human studies are still required to establish their diagnostic value and clinical utility.

For inorganic nanoprobes, clinical translation is governed not only by optical performance but also by their behavior in vivo. Biodistribution, biodegradation, clearance, and long-term biocompatibility are therefore critical considerations, particularly given the potential retention of poorly degradable materials in the liver and spleen [101]. Equally important is the reproducible manufacture of nanoprobes with well-defined size, composition, surface chemistry, and ligand density, as variations in these parameters can substantially alter both imaging performance and biological fate. These challenges become even more relevant for multifunctional platforms integrating diagnostic imaging with photothermal or photodynamic therapy, for which formulation, manufacturing, safety assessment, and regulatory evaluation are inherently more demanding.

AI may provide new opportunities to streamline probe discovery and optimization. Machine-learning approaches have already been applied to predict photophysical properties and guide fluorescent-probe design [29], while pattern-recognition algorithms have improved the classification of bacterial optical signatures generated by sensor arrays and targeted fluorescent probes [194]. Moving forward, AI could enable a more integrated design framework in which optical properties, bacterial specificity, physicochemical characteristics, biosafety, and performance in clinically relevant matrices are optimized simultaneously [195]. Such data-driven design, however, remains at an early stage for bacteria-targeted nanoprobes, largely because sufficiently large and standardized datasets linking probe structure to biological behavior and translational performance are still lacking.

Nevertheless, optical nanoprobes have already demonstrated feasibility in oncology, providing a roadmap for bacterial imaging. Emerging strategies to overcome current limitations include shifting to longer wavelengths (NIR-II/SWIR) or using photoacoustic and upconversion modalities to reduce scattering and autofluorescence, integrating optical probes with sample enrichment or microfluidic systems to achieve detection of low CFU levels, and designing biodegradable or cleavable probe chemistries with stealth surface coatings to improve clearance and minimize toxicity [196]. Overall, the exceptional sensitivity, versatility, and molecular precision of optical nanoprobes position them as transformative tools for real-time imaging of bacterial infections, with the potential to revolutionize early diagnosis, guide antibiotic stewardship, and advance precision therapy in infectious diseases.
